# Agile human activity recognition for wearable devices based on online incremental learning

**DOI:** 10.3389/fpubh.2026.1727388

**Published:** 2026-02-05

**Authors:** Lulu Fan, Hanyan Peng, Lei Xiao, Lang Shi, Siming Zhou, Yuyang Song, Huilong Fan

**Affiliations:** 1 Department of Hematology, Shanghai Changzheng Hospital, Shanghai, China; 2 Laboratory of Intelligent Collaborative Computing, University of Electronic Science and Technology of China, Chengdu, China; 3 School of Computer Science and Engineering, University of Electronic Science and Technology of China, Chengdu, China

**Keywords:** dynamic sparse subnetwork, edge computing, human activity recognition, online learning, venipuncture monitoring

## Abstract

**Background:**

Achieving high-precision, low-latency, and continuously adaptive human activity recognition on resource-constrained edge devices represents a core challenge. Existing research primarily focuses on improvements in single directions, such as “online learning,” “model sparsification,” or “feature extraction,” lacking a framework that synergistically optimizes all three. This leads to difficulties in dynamically balancing accuracy, latency, and power consumption when processing non-stationary sensor data streams.

**Methods:**

To address this, this paper designs an end-to-end closed-loop adaptive learning framework. The core innovation of this framework lies in its system-level synergistic design: (1) Employing fast principal component analysis for adaptive feature dimensionality reduction; (2) Introducing an information theory-based dynamic sparse subnetwork activation mechanism to tackle the NP-hard problem of model selection; and (3) Integrating a low-complexity online incremental learning module for real-time tracking of concept drift. Through the closed-loop feedback and control of the aforementioned components, this framework achieves joint dynamic optimization of feature extraction, model complexity, and adaptation speed under edge computing constraints.

**Results:**

Experimental results across five datasets demonstrate that this framework achieves accuracies ranging from 85.6% to 97.4%, with inference latency of approximately 1.0 ms.

**Conclusion:**

The framework comfortably meets the real-time requirement.

## 1 Introduction

Real-time monitoring of complex human activities (such as clinical venipuncture) through wearable sensors is essentially a dynamic optimization problem under strict resource constraints (1, 2). It not only requires algorithms to possess high classification accuracy and extremely low inference latency but also must adapt to the inherent high-dimensional and nonstationary characteristics of sensor data streams, i.e., effectively handling continuously occurring “concept drift” (3).

Currently, academia primarily pursues breakthroughs along three technical paths: designing lightweight static models (4, 5), utilizing network sparsification to compress models (6), and exploring online or continual learning algorithms (7, 8). However, these studies are largely isolated. Static models, once deployed, cannot adapt to changing conditions; sparsification techniques enhance efficiency but retain inherent static defects in models; online learning mechanisms often fail to meet millisecond-level latency requirements due to high computational overhead. A fundamental limitation is that existing methods fail to synergistically integrate the three key elements—“efficient feature extraction,” “dynamic model structure adjustment,” and “online parameter update”—into a unified, resource-aware closed-loop system. This “fragmented” research paradigm makes it difficult for systems to intelligently balance feature fidelity, computational sparsity, and learning stability in dynamic environments, thereby preventing the achievement of long-term, robust autonomous intelligence on edge devices.

This critical analysis unequivocally reveals a profound gap within the current body of research: the conspicuous absence of a unified mathematical framework capable of seamlessly integrating foundational principles derived from statistical signal processing, combinatorial optimization theory, and adaptive online learning into a cohesive, closed-loop system expressly engineered for the demanding constraints of edge computing platforms. Such an integrative framework is indispensable for intelligently navigating the intricate, often competing trade-offs inherent in real-time embedded intelligent systems, including the tension between preserving high-fidelity feature representations, enforcing extreme computational sparsity for efficiency, and ensuring provable stability in parameter convergence amid persistently dynamic operational landscapes.

To address this gap, we propose a novel framework that orchestrates three synergistic components. First, we employ a computationally efficient feature extraction module to create a robust data representation that is resilient to noise and input variations. Second, a dynamic subnetwork activation strategy adaptively allocates computational resources to dynamically balance inference accuracy against latency. Third, an online incremental learning mechanism ensures long-term model robustness by continuously adapting to the non-stationary data streams mentioned earlier. The entire system is stabilized and optimized by a closed-loop feedback controller that manages the interplay between these components.

In summary, this research establishes a pioneering cross-layer optimization paradigm for realizing adaptive intelligence on severely resource-constrained edge platforms. Its principal contributions are multifaceted: First, we establish a comprehensive methodological framework that unifies critical mathematical disciplines into a structured, theoretically grounded approach for developing edge-native adaptive systems. Second, we derive a suite of practical, low-complexity algorithms supported by strong theoretical guarantees encompassing approximation bounds and provable convergence properties, transforming the paradigm into implementable technology. Finally, we deliver exhaustive empirical validation demonstrating that our approach achieves accuracies ranging from 85.6% to 97.4% across five datasets, with inference latency of approximately 1.0 ms.

## 2 Related work

Our work is positioned at the intersection of three key research areas in edge computing: the design of efficient static models, the theory and application of network sparsity, and the challenge of adaptive online learning. This section reviews the state of the art in these domains to precisely situate our contribution.

### 2.1 Efficient static models for edge inference

The core idea of efficient static model methods is to develop computationally efficient models under the assumption of stationary data distributions, thereby enabling low-cost edge inference. Representative approaches include **CNN** (9) as the foundational framework for temporal processing, whose fixed structure precludes dynamic adjustment of computational load; **DeepConvLSTM** (10), which integrates CNN and LSTM to capture temporal dependencies, yet its recurrent architecture is computationally intensive and struggles to satisfy stringent latency constraints; **iSPLInception** (11), which employs multi-branch convolutions to extract multi-scale features, but exhibits high model complexity and substantial overhead for edge deployment; and **HART** (12), based on self-attention mechanisms, which can model long-range dependencies, although its Transformer architecture's quadratic complexity is challenging to sustain in real-time edge scenarios.

These models are all grounded in empirical risk minimization, and their static nature renders them incapable of adapting to variations in the data generation process, thereby constituting a primary limitation for deployment in dynamic edge environments.

### 2.2 Sparsity and model compression

The primary idea of sparsity and model compression methods is to reduce the number of parameters and operations in deep neural networks through network sparsification and model compression, thereby lowering inference computational costs and enhancing the efficiency of edge devices. Typical methods are based on the lottery hypothesis, which posits that densely randomly initialized networks contain sparse subnetworks that can be trained independently to high accuracy (6), thereby inspiring a series of pruning and sparsification techniques, such as static sparse subnetworks, used for offline identification of efficient substructures. Although these methods successfully reduce inference overhead, the generated models remain static and cannot handle non-stationary data, which has prompted the field to evolve toward dynamic sparse methods (13, 33, 34).

### 2.3 The challenge of adaptivity: online and continual learning

The core idea of adaptive online learning methods is to develop models capable of incrementally learning new data while retaining old knowledge, to address the challenges of concept drift in data streams, thereby achieving long-term robust deployment of machine learning systems in the real world. Typical methods include the use of experience replay and parameter regularization to avoid catastrophic forgetting (7), exploring various continual learning strategies in human activity recognition, but often facing a trade-off between plasticity and stability (8, 35, 36). Existing online learning mechanisms typically focus on weight updates, requiring substantial computational resources for retraining or backpropagation, rendering them unable to meet the demands of applications with millisecond-level latency constraints.

This reveals the critical research gap that our work aims to fill. There is a lack of a unified, mathematically-grounded framework that co-optimizes the entire adaptive pipeline on an edge device in a closed-loop fashion. Specifically, few studies have integrated adaptive feature extraction, dynamic model structure selection, and low-complexity online parameter updates into a single, cohesive system orchestrated by a formal resource-aware control mechanism. Our paper proposes such a framework, providing a principled approach to building truly adaptive and efficient edge intelligence systems.

## 3 Problem model

### 3.1 System model

To enable real-time and precise monitoring of venipuncture procedures, the study designs a lightweight edge intelligence system. The system architecture comprises two primary components: a **smart wearable device** and an **edge computing node**. The wearable device, equipped with gyroscope sensors, captures real-time data (*X*(*t*)) that reflects the practitioner's hand stability during the puncture process. This data is transmitted to the edge computing node for analysis.

The edge node hosts the core intelligence of the system, which consists of three synergistic software modules. The data processing pipeline commences with a **Feature Extraction** module that generates a low-dimensional feature vector, **f**∈ℝ^*d*^. This vector is fed into a **Dynamic Sparse Subnetwork Activation** module, which employs a lightweight convolutional neural network for classification. The module dynamically selects a weight matrix **W**_*c*_ based on the features **f** to produce the classification output, *y*. Finally, an **Incremental Learning** module continuously monitors anomalous behaviors, enabling rapid online model updates. This framework establishes a complete end-to-end data flow:


$$\begin{array}{c} \text{Sensor Data }(X(t))\to \text{Feature Vector }(f)\to \text{Dynamic Sparse}\\ \text{Subnetwork Activation}(\text{LCNN})\to \text{Dynamic Classification}(y)\to \\ \text{Model Update \& Feedback}. \end{array}$$

Among these, LCNN denotes Lightweight Convolutional Neural Network.

### 3.2 Problem definition

Given the raw sensor data stream *X*(*t*) from a practitioner's operation and the complete set of network parameters **W**, the primary objective of this study is to develop an optimal strategy for dynamic sparse subnetwork activation. This strategy requires real-time selection of an ideal weight subset ${\text{W}}_{c}^{*}\subset \text{W}$ to maximize monitoring accuracy while minimizing inference latency. Formally, this task constitutes a combinatorial optimization problem. The discrete nature of parameter selection renders it NP-hard, akin to the classic 0–1 knapsack problem, thereby posing a substantial theoretical challenge.

#### 3.2.1 Operating constraints

The search for the optimal subnetwork is subject to practical constraints. Any viable solution must adhere to the stringent requirements of clinical monitoring on resource-constrained edge devices.

The solution must meet rigorous constraints on performance and resources: the inference latency of any subnetwork, *T*_infer_(**W**_*c*_), does not exceed the maximum threshold *T*_max_ to ensure real-time responsiveness; its power consumption, *P*(**W**_*c*_), remains within the device's budget *P*_max_; and the classification accuracy on the current data window, Acc(**W**_*c*_, *X*(*t*)), is maintained above the clinically acceptable minimum Acc_min_. To ensure computational efficiency, the model is subject to two sparsity constraints: the number of activated parameters (denoted by binary variables *a*_*i*_) does not exceed the total budget *K*, where *K* is significantly smaller than the total number of parameters in the full model; and the dimension of the input feature vector, dim(**f**), is limited to a maximum of *d*_max_.

Finally, the system's adaptability is time-constrained, requiring that the time for any online model update, *T*_update_, be completed within the maximum duration *T*_*u*, max_.

#### 3.2.2 Objective function

The objective function is a multi-objective optimization function combining accuracy and inference latency, defined using the weighted sum method:


$$F\left({\text{W}}_{c}\right)=\alpha \frac{T_{\text{infer}}\left({\text{W}}_{c}\right)}{T_{\text{max}}}-\beta \text{Acc}\left({\text{W}}_{c},X\left(t\right)\right),$$

where α, β are weighting coefficients satisfying α+β = 1. We formulate the optimization objective as:


$$\underset{{\text{W}}_{c}}{\min}F\left({\text{W}}_{c}\right).$$

The practical significance is to achieve the optimal trade-off between nursing monitoring accuracy and inference latency under the constraints of edge computing resources.

### 3.3 Computational complexity analysis

The problem of selecting an optimal sparse subnetwork, as described in the previous section, belongs to the class of combinatorial optimization problems. In particular, the task of selecting a subset of parameters to maximize accuracy under a sparsity constraint directly parallels the classical 0–1 Knapsack Problem.

A fundamental result in computational complexity theory establishes that the 0-1 Knapsack Problem is NP-hard (14). This implies that, unless P = NP, no polynomial-time algorithm exists to compute the exact optimal solution relative to the number of parameters. Proofs typically involve reductions from other known NP-complete problems, such as the Partition Problem (15). Consequently, by reduction, the dynamic subnetwork activation problem defined in this paper is also NP-hard. This classification emphasizes the necessity of the efficient heuristic and approximation algorithms presented in Section 4, as exact solutions prove computationally infeasible for networks of practical scale.

## 4 Methods

This section details our proposed adaptive edge computing framework. This framework addresses the core challenge of real-time monitoring of procedures such as venipuncture on resource-constrained devices.

To address the real-time demands of resource-constrained devices and the challenges of processing high-dimensional noisy signals, we propose a four-layer collaborative architecture guided by information-theoretic optimality and closed-loop feedback control principles. First, an orthogonal feature-extraction module is designed to address the inefficient representation of noisy raw signals (Section 4.1). Subsequently, to tackle the NP-hard problem of model selection, a dynamic subnetwork is developed that adaptively matches each input sample with a sparse model, achieving an optimal trade-off between computational efficiency and classification performance (Section 4.2). Next, an online adaptive learning module is introduced to handle concept drift in dynamic environments (Section 4.3). Finally, the framework integrates all functional components into a unified end-to-end architecture. Through a closed-loop feedback control system, it dynamically adjusts computational resources in real time to sustain high performance under stringent latency constraints (Section 4.4).

**Figure 1:**
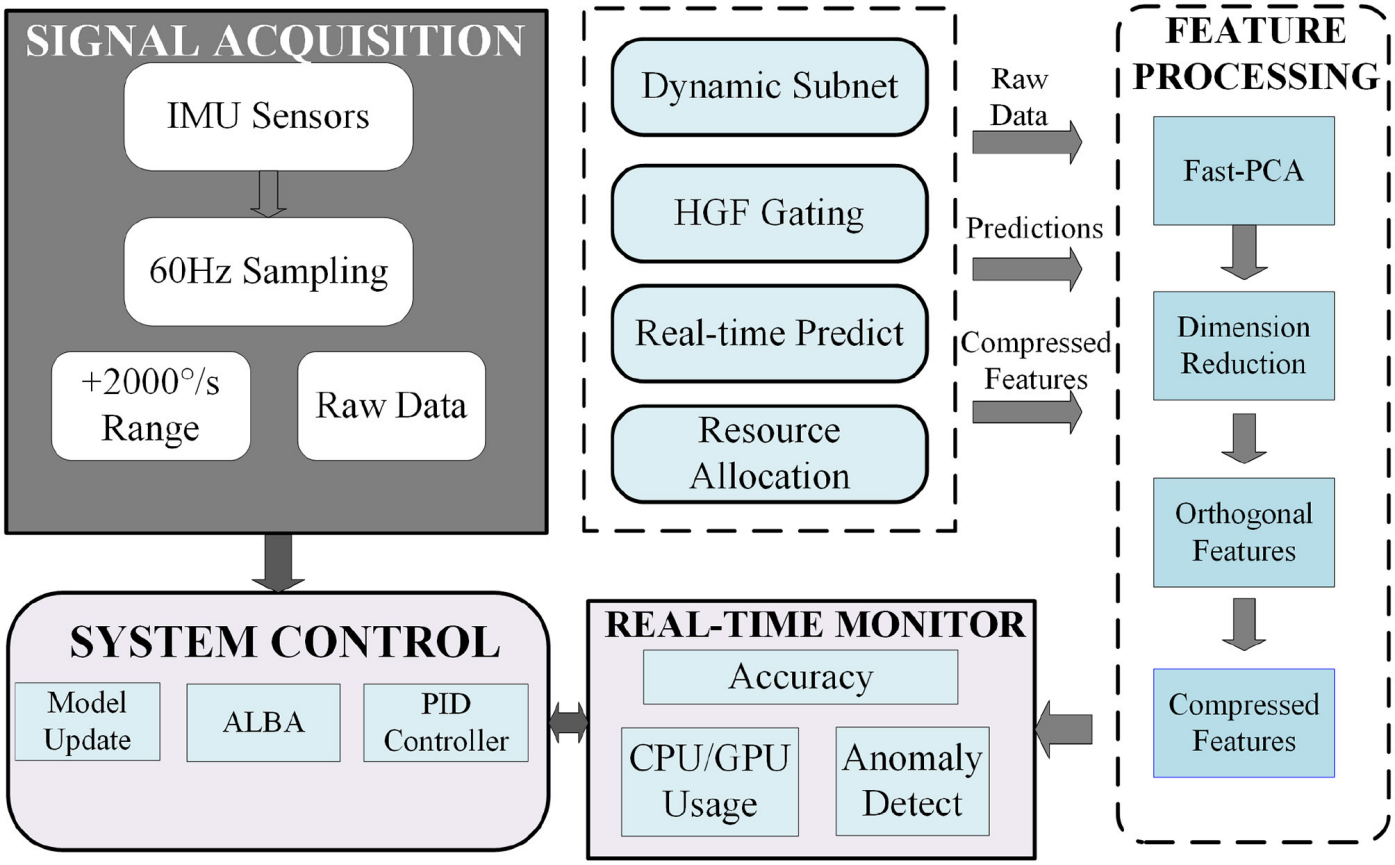
System architecture of the proposed closed-loop adaptive framework for real-time edge intelligence.

To elucidate the implementation details of this framework, Figure 1 illustrates its overall architecture. This highly integrated closed-loop system consists of five core modules that collaborate to form a complete processing pipeline: (1) the Signal Acquisition module for raw data collection, (2) the Feature Processing pipeline for efficient representation learning, (3) the Core Inference Engine for dynamic subnetwork activation, (4) the Real-time Monitor for performance evaluation and anomaly detection, and (5) the System Control module for feedback regulation and adaptation. The subsequent subsections provide a detailed exposition of the design of each core component, in accordance with the presented logical architecture.

### 4.1 Orthogonal feature extraction module

Building accurate and efficient data representations is fundamental. However, raw multimodal sensor data from gyroscopes, accelerometers, and magnetometers inherently exhibit high dimensionality, complex noise, and strong feature coupling. In particular, cross-channel correlations arising from the nonlinear dynamics of human motion make this data difficult to use directly for real-time analysis. Inputting such high-dimensional, highly correlated data without preprocessing results in an exponential increase in computational load, making real-time inference challenging to meet the latency constraints of edge devices.

**Figure 2:**
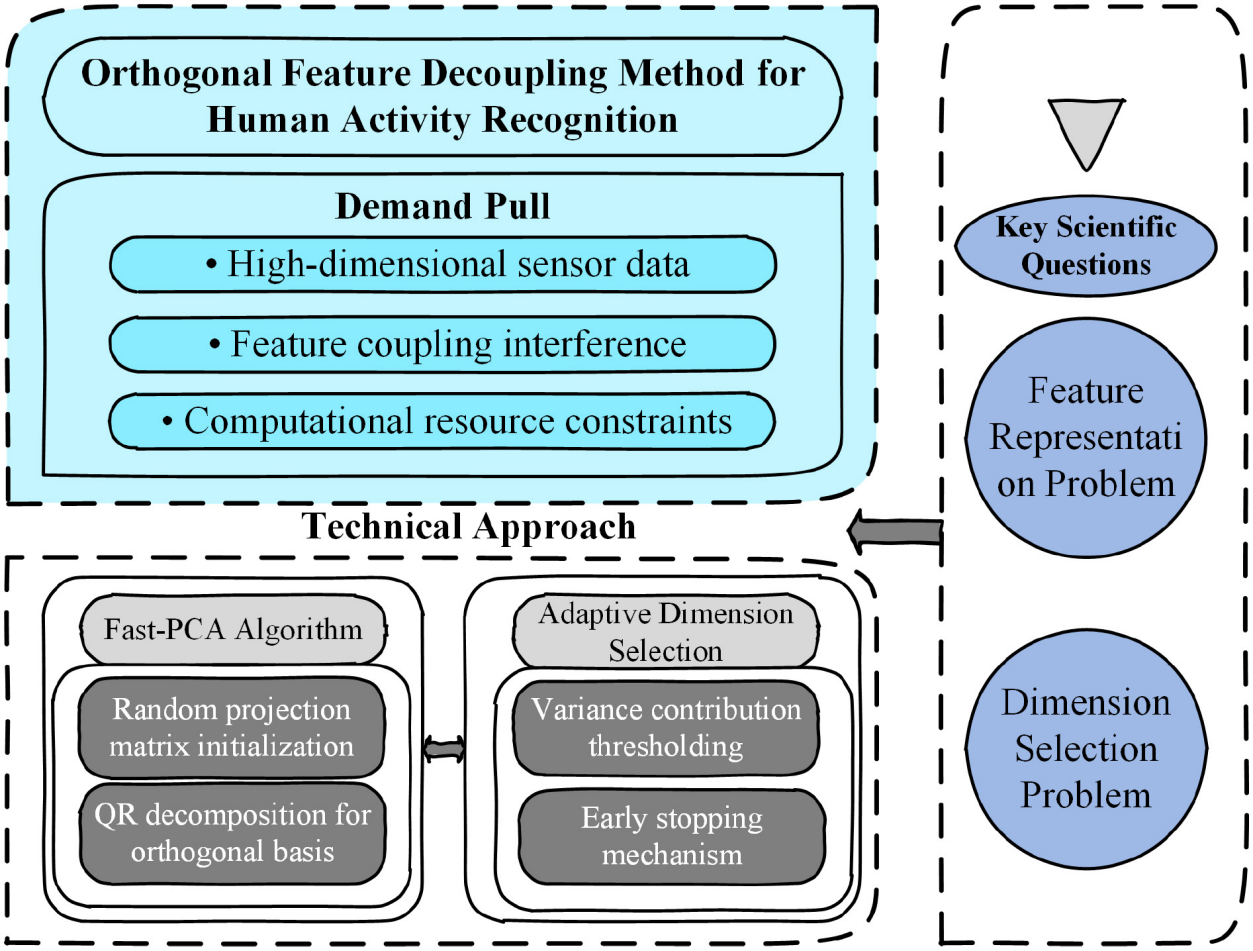
Conceptual framework for the orthogonal feature decoupling method, outlining the demand pull, key scientific questions, and the proposed technical approach.

To address this challenge, this section proposes a feature representation method based on an improved form of principal component analysis, termed Fast-PCA. This approach substantially reduces computational complexity while retaining the essential property of conventional PCA—namely, its capability to decorrelate feature dimensions. The overall conceptual framework, illustrated in Figure 2, consists of two main components: the feature projection mechanism (Section 4.1.1) and the adaptive criterion for determining the optimal number of retained features (Section 4.1.2).

#### 4.1.1 Feature projection via fast-PCA

Although traditional PCA effectively extracts principal components, its reliance on the full covariance matrix's singular value decomposition results in an *O*(*n*^3^) computational complexity, making it difficult to deploy in resource-constrained environments. Therefore, this section proposes a Fast-PCA projection method based on stochastic numerical linear algebra. By integrating statistical feature decoupling and adaptive dimension selection, this approach reduces computational complexity to *O*(*n*^2^*d*) while supporting dynamic optimization of the feature space and preserving feature orthogonality.

To accommodate real-time monitoring scenarios, this method applies short-time sliding window processing to sensor streams. The framework relies on the assumption that within sufficiently short time windows, the second-order statistical properties of the signal remain approximately stable. Consequently, each window can be treated as an independently modelable data block. Let the sliding window length be *T*. The n-dimensional raw signal output from the sensor forms a matrix within the window:


$$\begin{array}{l} \text{X}={\left[{\text{x}}_{\left(t-T+1\right)},{\text{x}}_{t-T+2},\ldots ,{\text{x}}_{t}\right]}^{T}\in {\mathbb{R}}^{T\times n}, \end{array}$$ (1)

where ${\text{x}}_{i}\in {\mathbb{R}}^{n}$ is the raw gyroscope signal vector at time *i*.

Furthermore, we assume that sensor data possesses an implicit low-dimensional structure. Despite the high original dimension n, the most useful discriminative information is concentrated in a small number of principal directions. Therefore, our goal is to find an orthogonal projection that maps the data to a much smaller dimension, *d*≪*n*, while ensuring the cumulative variance explained by these components meets a minimum threshold, η_min_.

##### 4.1.1.1 Fast covariance estimation and eigen-decomposition

The computational bottleneck of traditional PCA lies in the need to construct and decompose an n × n covariance matrix. To address this challenge, we adopt the “sketch-and-solve” approach from randomized linear algebra. By constructing low-dimensional random sketches to approximate the covariance structure of the original space, we reduce the overall complexity to *O*(*n*^2^*d*). This process involves two key steps:

**Step 1: Randomized sketching** First, instead of directly analyzing X, we follow the principles of random projection theory (16) to compress it into a smaller subspace using a random Gaussian matrix. From this, we generate an orthogonal basis that preserves the primary variance information. This step avoids recalculating the original high-dimensional matrix and serves as the primary acceleration source for the entire method.

**Step 2: Decomposition on the sketch** With this compact basis **Q** in hand, we project the original data onto it to form a much smaller matrix, **B** = **Q**^*T*^**X**. This *k*×*n* matrix contains a compressed representation of the original data's covariance structure. We then apply an efficient iterative method, such as the Lanczos bidiagonalization algorithm (17), to compute the Singular Value Decomposition (SVD) of **B**. The principal components and corresponding eigenvalues of the original data **X** can be accurately recovered from this decomposition. The final projection matrix **P** is constructed from the resulting right singular vectors **V**:


$$\begin{array}{l} \text{P}=\text{V}\left(:,1:d\right), \end{array}$$ (2)

where **V** contains the right singular vectors derived from the SVD of **B**.

This process ensures that all computations are performed in a randomly projected subspace of significantly lower dimension than the original, thereby avoiding direct processing of the covariance matrix.

Specifically, our Fast-PCA improvements include two key innovations: **(1) Adaptive sketch dimension tuning**. Replacing traditional Fast-PCA's fixed sketch size, we dynamically adjust it based on the cumulative variance threshold to avoid redundant computation or information loss; **(2) Decoupling-reorthogonalization integration**. Optimizing Fast-PCA's core process by combining statistical feature decoupling with Lanczos bidiagonalization, using partial instead of full reorthogonalization to reduce SVD iteration costs while preserving feature orthogonality.

This approach significantly reduces computational complexity. The total complexity of Fast-PCA can be expressed as the sum of two terms:


$$C=O\left(Tnk\right)+O\left(k^{2}m\right),$$

where *k* is the sketched dimension and *m* is the number of Lanczos iterations. When *k* = *O*(*d*) and *m* = *O*(*d*), the total complexity effectively reduces to *O*(*n*^2^*d*), achieving an order-of-magnitude optimization compared to the *O*(*n*^3^) complexity of traditional PCA.

##### 4.1.1.2 Adaptive feature selection criterion

The efficiency of the Fast-PCA algorithm requires integration with an intelligent selection of the feature dimension d^*^ to perform effectively in resource-constrained environments. This section thus introduces an adaptive mechanism: a criterion that adjusts the target dimensionality according to real-time computational load, thereby facilitating a dynamic and principled trade-off between the richness of the feature representation and processing latency.

The dynamic variance contribution ratio threshold η(*t*) is defined to determine the minimal effective dimension d^*^ through the optimization problem:


$$\begin{array}{l} d^{*}=\min\left\{d^{\prime }\in {\mathbb{N}}^{+}|\frac{\overset{d^{\prime }}{\underset{i=1}{\sum }}\lambda_{i}}{\text{tr}\left(\Sigma \right)}\geq \eta \left(t\right)\right\}. \end{array}$$ (3)

This constraint ensures the cumulative variance contribution of selected features meets the current threshold. The threshold η(*t*) adapts in real-time based on computational resources:


$$\begin{array}{l} \eta \left(t\right)=\eta_{\max}-\alpha \cdot \frac{T_{\text{used}}}{T_{\max}}, \end{array}$$ (4)

where α is a decay coefficient, and *T*_used_ is the computation time consumed within the current processing window.

This randomized approach is theoretically well-founded. Established results in randomized numerical linear algebra (16) demonstrate that the approximation error of the randomized SVD, which underlies our Fast-PCA method, possesses a rigorous theoretical bound. Specifically, the expected projection error is dominated by the magnitude of the largest discarded singular value, σ_*r*+1_(**X**). This guarantee ensures that the energy or information corresponding to the lost features during projection is negligible, thereby validating that the resulting low-dimensional feature space achieves a high-fidelity representation of the original signal.

##### 4.1.1.3 Algorithm implementation

The pseudocode implementation for our Fast-PCA with the adaptive selection mechanism is presented in Algorithm 1.

Algorithm 1 Fast-PCA with adaptive dimension selection. 
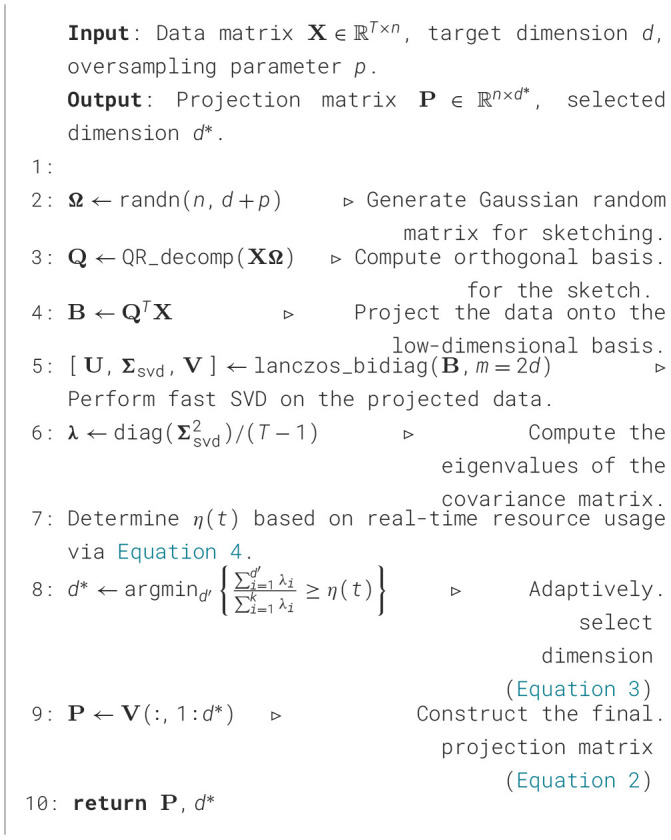

#### 4.1.2 Dynamic adaptive feature selection

Although the Fast-PCA mechanism in Section 4.1.1 effectively achieves dimensionality reduction, determining the optimal target dimension d^*^ remains challenging. Fixed dimensionality schemes prove inadequate due to complexity variations across venipuncture phases, while simultaneous constraints of real-time processing (*T*_max_ ≤ 5 ms) and edge device capacity (*d*_max_ ≤ 6) necessitate dynamic dimensionality adaptation. However, recomputing the full eigenspectrum for variance contribution analysis at each iteration imposes prohibitive computational overhead, fundamentally compromising Fast-PCA's efficiency advantages.

To address this issue, this section proposes an adaptive selection criterion based on the variance contribution rate. By integrating variance energy distribution, real-time resource awareness thresholds, and rapid update strategies, it achieves dynamic dimension determination with minimal computational overhead. Its core principle is to leverage the energy concentration of PCA by monitoring the cumulative variance contribution rate of the preceding principal components to determine the required feature dimensions for the current window. For principal components sorted by eigenvalue in descending order, the cumulative variance contribution rate is defined as:


$$\begin{array}{l} \Gamma_{j}=\frac{\overset{j}{\underset{i=1}{\sum }}\lambda_{i}}{\overset{d}{\underset{i=1}{\sum }}\lambda_{i}}, \end{array}$$ (5)

where λ_*i*_ denotes the *i* eigenvalue and Γ_*j*_ measures the coverage of the first *j* principal components over the total variance. Multiple physiological signals exhibit a structure of rapidly declining eigenvalues, typically showing a distinct inflection point after the first few principal components, causing subsequent components to contribute minimally to the overall variance. Therefore, the smallest *j* satisfying the cumulative variance contribution reaching a specified threshold can be regarded as the effective dimension for the current window.

However, in practical systems, maintaining a fixed threshold can create performance bottlenecks when signal complexity or computational resources change: during resource constraints, high thresholds lead to excessive dimensionality, causing delay overruns, while low thresholds under ample resources prematurely discard potentially valuable information. To address this, a dynamically varying threshold function η(*t*) is introduced. This function aims to reflect the system's remaining time and resource utilization in real time, enabling the dimension reduction process to automatically contract or expand the representation space based on the current environment. This threshold is defined as:


$$\eta \left(t\right)=\eta_{\min}+\frac{\alpha }{1+e^{-\beta \left(T_{\max}-T_{\text{used}}\left(t\right)\right)}}\;\left(\alpha >0,\beta >0\right).$$

Here, *T*_used_(*t*) denotes the computational time already consumed by the current window, where *T*_max_ represents the maximum allowable time limit for the entire processing workflow. When sufficient time remains, the function output increases, favoring the retention of more principal components to enhance feature expression capabilities. As time progressively depletes, the function value gradually decreases, making the dimensionality reduction process more conservative to ensure the system completes computations before the deadline.

##### 4.1.2.1 Fast iterative covariance update strategy

The dimensionality selection process achieves real-time performance largely due to the efficient update mechanism of PCA spectral information. In sliding window mode, each window shift only introduces a low-rank perturbation to the covariance matrix—specifically, adding one new sample and removing one old sample. This property enables rapid updates to eigenvalue changes using linear approximation methods, eliminating the need for repeated complex matrix decomposition operations. The computation required for such linear updates relies solely on vector-level operations, with an overall complexity of *O*(*d*), enabling millisecond-level real-time processing. The updated sequence of eigenvalues continues to reflect the energy ranking structure of principal components, ensuring stable and reliable calculation of cumulative variance contribution.

##### 4.1.2.2 Dual-threshold triggering mechanism

To balance computational efficiency with decision stability, we propose a dimension adjustment strategy based on a dual-threshold mechanism. This approach combines a fixed, conservative baseline with a dynamic, adaptive criterion. The foundation of this mechanism is a **hard threshold**, η_min_, which ensures that the cumulative variance contribution rate never falls below a clinically acceptable lower limit (e.g., η_min_ = 0.95), thereby guaranteeing a minimum level of feature quality. To introduce adaptability, we augment this with a **soft threshold**, η_soft_(*t*), which is dynamically adjusted based on the real-time resource load. It is defined as:


$$\begin{array}{l} \eta_{\text{soft}}\left(t\right)=\eta_{\min}+\delta \cdot \left(1-\frac{T_{\text{used}}\left(t\right)}{T_{\max}}\right),\;\left(0<\delta <1-\eta_{\min}\right). \end{array}$$ (6)

This soft threshold allows the system to be more demanding on feature quality (higher η) when computational time is abundant and more lenient (lower η) when the system is under temporal pressure.

The final dimension decision rule is:


$${d^{\prime }}^{*}=\max\left\{\min\{j|\Gamma_{j}\geq \eta_{\min}\},\min\{j|\Gamma_{j}\geq \eta_{\text{soft}}(t)\}\right\}.$$ (7)

This rule employs a conservative dimensionality strategy to improve accuracy under sufficient resource conditions and an aggressive dimensionality reduction strategy to guarantee real-time performance under constrained resources. The effect of this decision rule on classification performance is grounded in established statistical learning theory (18), which delineates a direct relationship between the retained variance of features and the classification error rate. Specifically, it ensures that if the proportion of variance preserved by the selected features is adequate, the classification error resulting from dimensionality reduction possesses a well-defined and controllable theoretical upper bound. This principled framework guides the setting of a minimum threshold η_min_, thereby maintaining system performance at an acceptable level even in the most stringent resource-constrained scenarios.

##### 4.1.2.3 Fast sorting and early stopping algorithm

To avoid the (*O*(*d*log*d*)) cost of fully sorting all eigenvalues, we exploit their monotonic decay to design a linear-time early-stopping rule. The key idea is that once the cumulative variance at index *j* reaches the target level Γ_*j*_≥η_min_, and the remaining eigenvalues are sufficiently small, the cumulative variance will remain above the threshold for all subsequent indices.

This condition provides a computationally efficient check, as stated in the following theorem:

Theorem 1. If the (*j*+1)-th eigenvalue satisfies


$$\lambda_{j+1}\leq \frac{\left(1-\eta_{\min}\right)\overset{d}{\underset{i=1}{\sum }}\lambda_{i}}{d-j},$$

then the cumulative variance contribution satisfies Γ_*j*_≥η_min_.

This result follows from the monotonicity of eigenvalues, which ensures that the tail sum $\overset{d}{\underset{i=j+1}{\sum }}\lambda_{i}$ is bounded by (*d*−*j*)λ_*j*+1_.

Thus, meeting these two conditions at the index *j* guarantees that the threshold is safely satisfied, allowing the search to terminate without examining the remaining components. This yields an *O*(*d*) early-stopping algorithm that preserves the correctness of the full sorting approach while significantly reducing computational cost.

##### 4.1.2.4 Algorithm implementation

This entire adaptive process, achieving feature selection in *O*(*d*) complexity, is implemented in Algorithm 2.

Algorithm 2 Adaptive dimension selection with early stopping. 
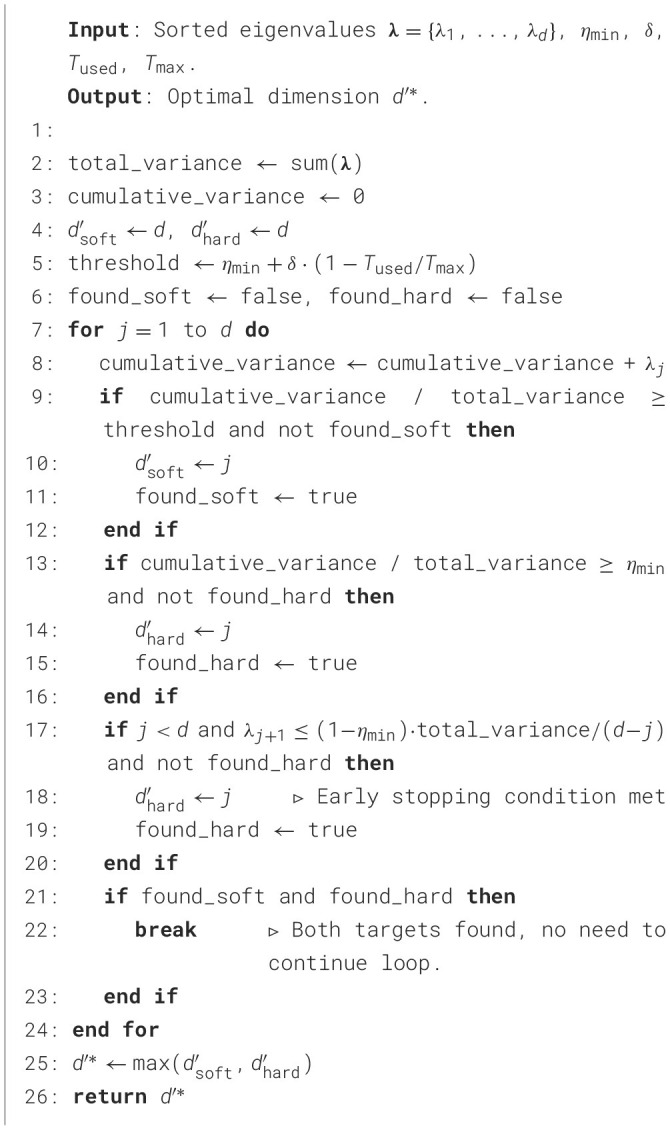

The design of Algorithm 2 focuses on maximizing computational efficiency while maintaining robust and adaptive decision-making. Key design strategies are employed: first, the pre-sorted eigenvalues **λ** from the PCA process are leveraged to eliminate the costly *O*(*d*log*d*) sorting step, thereby enhancing efficiency; second, a dynamic soft threshold (Line 4) is introduced, computed once per invocation, to adaptively adjust feature quality requirements based on real-time computational resource availability, improving adaptability.

The core of the algorithm lies in the main loop (Lines 8–18), which is optimized via a dual early stopping mechanism. This mechanism integrates a hard threshold and the condition from Theorem 1, enabling loop termination as soon as a suitable dimension is identified to prevent unnecessary iterations. Finally, the decision rule (Line 21) takes the maximum of the dimensions determined by the two thresholds, ensuring a minimum quality level ($d_{\text{hard}}^{\prime }$) while striving for higher fidelity ($d_{\text{soft}}^{\prime }$) when resources permit, thus achieving a robust balance between feature fidelity and real-time performance.

### 4.2 Dynamic sparse subnetwork activation strategy

Building upon the low-dimensional yet informative feature representations developed in Section 4.1, this paper addresses the critical challenge of real-time classification under stringent resource constraints by proposing a dynamic sparse subnetwork activation strategy. This approach intelligently tailors computational pathways to achieve an optimal balance between efficiency and accuracy.

Conventional static models employ fixed computational architectures that cannot adapt processing load according to input complexity, leading to suboptimal resource utilization. To overcome this, we formulate the optimal subnetwork selection as a combinatorial optimization problem in parameter space, which exhibits NP-hard complexity analogous to the 0–1 knapsack problem. This manifests in two key aspects: (1) the computational intractability of exhaustive search methods and (2) the inadequacy of simple greedy approaches to deliver satisfactory suboptimal solutions within millisecond-level latency constraints.

**Figure 3:**
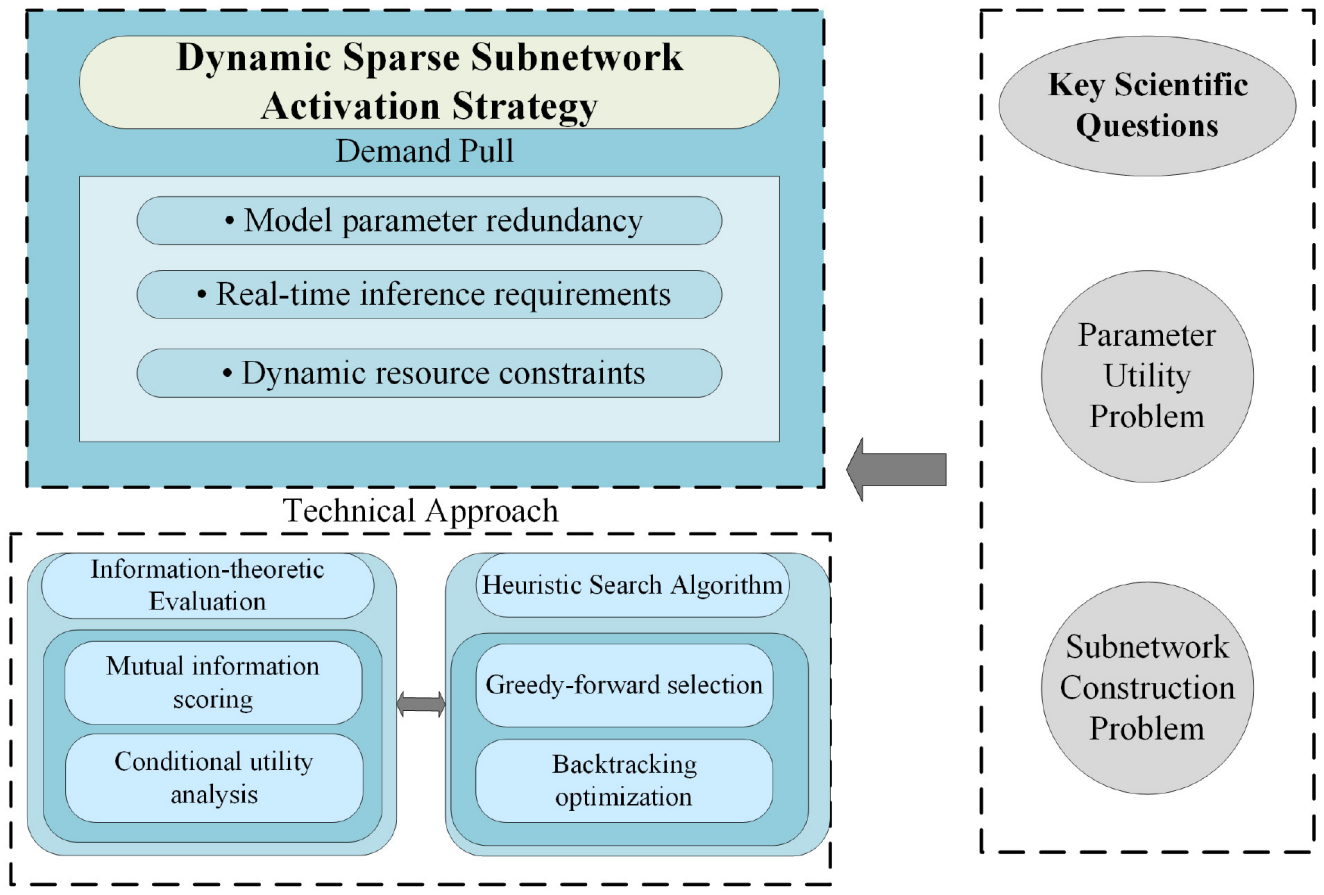
Conceptual framework for the dynamic sparse subnetwork activation strategy, outlining the demand pull, key scientific questions, and the proposed technical approach.

Our innovative solution transforms this intractable optimal subnetwork search into a computationally feasible heuristic optimization for maximum information gain. Grounded in information-theoretic principles, the strategy selectively activates the most discriminative network parameters for each input sample. As illustrated in Figure 3, our technical framework comprises three key components:

First, we establish theoretical foundations through information-gain-based parameter selection criteria, providing rigorous justification for dynamic subnetwork activation (Section 4.2.1).

Second, we develop dual implementation mechanisms: (1) a rapid gradient-aware gating function for real-time activation (Section 4.2.2) and (2) a dynamic weight adjustment method based on competitive neural networks (Section 4.2.3) (19).

#### 4.2.1 Dynamic parameter selection mechanism based on heuristic information gain

The activation of a dynamic sparse subnetwork is formalized as the identification of an optimal parameter subset within a vast combinatorial space $\Theta =\left\{{\text{W}}_{c}\in {\mathbb{R}}^{m\times d}|||\text{vec}\left({\text{W}}_{c}\right)|{|}_{0}\leq K\right\}$. The search space size, of order *C*(|**W**|, *K*), can exceed 10^6^, posing a significant challenge. Conventional greedy algorithms exhibit *O*(*K*|**W**|) complexity, which is infeasible under strict real-time constraints and fails to account for coupled information effects among features.

To address this, we propose a heuristic search strategy grounded in information theory, transforming parameter selection into a tractable problem of information gain maximization. Specifically, the utility of activating a parameter *w*_*j*_ is quantified by its **conditional mutual information** *I*(*f*_*j*_; *y*∣**f**_*S*_), measuring the novel information about the label *y* contributed by feature *f*_*j*_ given the selected feature set **f**_*S*_. This approach yields a near-optimal solution with substantially reduced complexity, approximately *O*(*K*log|**W**|).

The viability of this strategy hinges on two key properties: computational feasibility and theoretical soundness.

**Computational feasibility**: Real-time mutual information computation is enabled by assuming local conditional independence among features, allowing efficient information gain estimation without succumbing to the “curse of dimensionality.”

**Theoretical soundness**: We assume the utility function exhibits sub-modularity (20), which formalizes the “diminishing returns” property: the marginal information gain from adding a parameter to a smaller set is no less than that from adding it to a larger set. This ensures that a simple greedy algorithm achieves a solution with a provable guarantee—no worse than (1 − 1/*e*) of the true optimum.

##### 4.2.1.1 Fast conditional mutual information estimation

Direct mutual information computation requires joint probability distribution estimation with *O*(*Cd*^2^) complexity. For real-time feasibility, we introduce an incremental estimation method based on Kernel Density Estimation (KDE).

The reliability of this approach is ensured by the strong convergence properties of KDEs. As the sample size *N* increases and the kernel bandwidth *h* is reduced within an appropriate range, the density estimate $\hat{p}\left(f_{j},y\right)$ provided by the KDE will converge to the true distribution *p*(*f*_*j*_, *y*) almost everywhere (21). It follows that entropy-related measures based on this density—including mutual information—will also approximate their true values in a probabilistic sense.

Under the aforementioned convergence conditions, the upper bound on the estimation error of mutual information can be described as:


$$\begin{array}{l} |I\left(f_{j};y\right)-Î\left(f_{j};y\right)|\leq O\left(\frac{1}{\sqrt{N}h}\right)+O\left(h^{2}\right), \end{array}$$ (8)

The kernel bandwidth *h* can be selected using Silverman's empirical rule. The two error components in the above equation correspond to the variance term arising from finite sample size and the bias term introduced by kernel smoothing. When *Nh* → ∞ is satisfied, both gradually diminish, thereby ensuring the stability and accuracy of the estimation.

Overall, by leveraging the statistical convergence properties of KDE and its entropy estimation, this method achieves computationally efficient and theoretically grounded rapid estimation of conditional mutual information without requiring high-dimensional density modeling.

##### 4.2.1.2 Hierarchical pruning heuristic search

We model the parameter selection as a constrained sub-modular function maximization problem:


$$\begin{array}{l} \underset{S\subseteq \text{W}}{\max}U\left(S\right)\;\text{subject to }|S|\leq K. \end{array}$$ (9)

Where *U*(*S*) denotes the information-based utility of a parameter set. Solving this problem exactly is computationally intractable due to its combinatorial nature. Therefore, we develop a hierarchical three-stage heuristic search strategy that exploits sub-modularity to improve efficiency.

In stage one, each parameter group undergoes independent utility evaluation, retaining the top 2*K* candidates with the highest utility. This step significantly reduces the search space while preserving most potentially valuable parameters. In stage two, perform a greedy search on the filtered parameter set, selecting the parameter group that yields the maximum conditional gain at each step—that is, the group with the highest marginal utility within the currently selected set. This phase features low computational complexity and stable performance, providing a robust initial solution. In stage three, to further enhance result quality, we introduce an improved lazy greedy backtracking method. This approach leverages the marginal diminishing properties of sub-modular functions to skip unpromising candidates during local search, thereby optimizing greedy solutions at minimal computational cost.

Due to the sub-modular property of the utility function, this method provides provable approximation quality. The utility of the resulting parameter subset *s* approximates that of the optimal set S^*^:


$$\begin{array}{l} U\left(S\right)\geq \left(1-1/e\right)U\left(S^{*}\right)-\epsilon , \end{array}$$ (10)

where ϵ denotes the truncation error introduced by the coarse-grained screening step. This bound demonstrates that the algorithm maintains a robust performance lower bound even under strong pruning conditions.

##### 4.2.1.3 Algorithm implementation

The complete process is formally outlined in Algorithm 3, which details the synergistic interaction of its core components.

Algorithm 3 Hierarchical pruning for dynamic activation. 
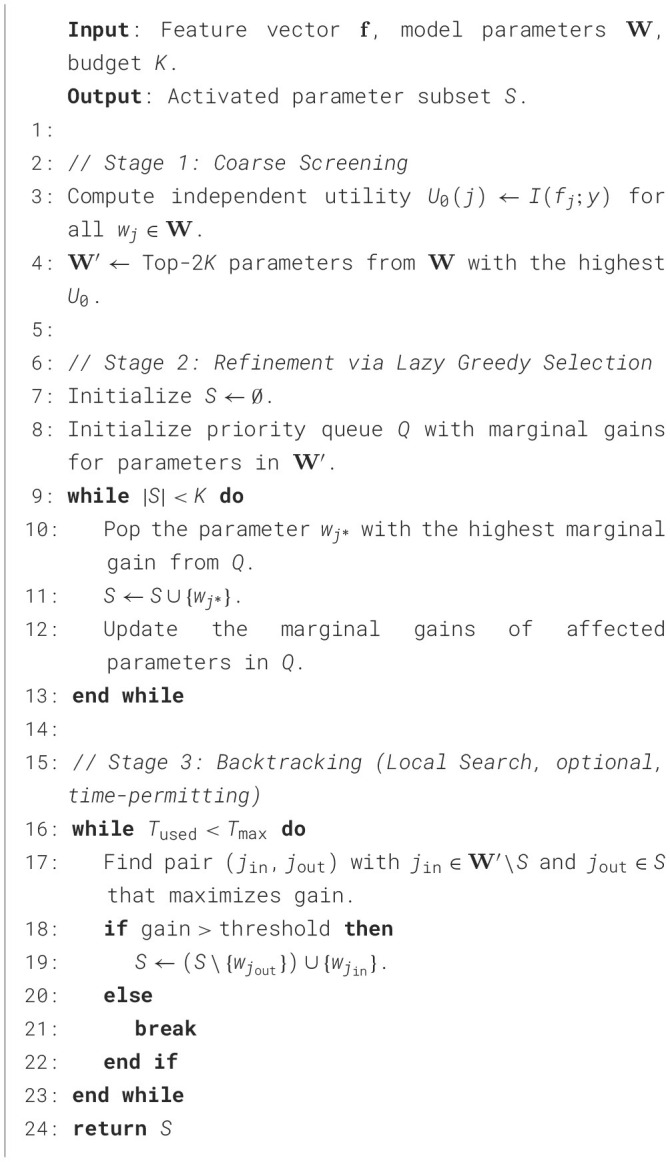

The design of Algorithm 3 aims to balance solution quality with strict real-time constraints. The algorithm is structured into three synergistic stages: coarse screening, refinement, and backtracking. The coarse screening stage (Lines 2–3) employs a computationally efficient metric (independent mutual information) to rapidly prune irrelevant parameters, reducing the search space to meet latency requirements. The refinement stage (Lines 6–12) utilizes a lazy greedy approach with a priority queue, which avoids redundant evaluations of marginal gains by leveraging sub-modularity, thereby reducing the complexity from *O*(*K*|**W**′|) to *O*(*K*log|**W**′|) for the selection phase. The backtracking stage (Lines 15–22) is an optional fine-tuning mechanism that executes only if residual time is available within the processing budget, enabling a dynamic trade-off between further improvement of solution quality and timely completion.

#### 4.2.2 Dynamic sub-network activation mechanism design

Section 4.2.1 establishes the theoretical foundation for parameter selection based on information gain; however, a practical and computationally efficient mechanism is imperative for real-time implementation. The essence of dynamic subnetwork activation resides in the design of such a mechanism. Conventional gating techniques, including soft masks, typically necessitate gradient retention for all parameters, thereby infringing the strict sparsity constraint ∑*a*_*i*_ ≤ *K* and compromising optimality guarantees.

To address these shortcomings, this section proposes a heuristic gating function (HGF). We model the activation decision as a constrained information gain maximization problem and prove that HGF furnishes a locally optimal solution. This method enables real-time activation decisions with *O*(1) complexity per parameter.

The proposed HGF determines the binary activation state *a*_*j*_∈{0, 1} for each parameter *w*_*j*_ by thresholding its importance score *s*_*j*_:


$$\begin{array}{l} a_{j}=I\left[s_{j}\geq \tau -\gamma \cdot \frac{\partial L}{\partial s_{j}}\right], \end{array}$$ (11)

where τ is the global baseline threshold, $\gamma \cdot \frac{\partial L}{\partial s_{j}}$ provides gradient-based modulation, and 𝕀[·] denotes the indicator function.

This dynamic thresholding mechanism adapts to sample difficulty: via the gradient term, the gate imposes less stringent constraints on high-loss samples.

The theoretical validity of this HGF design relies on two key assumptions:

First, parameter importance scores must remain stable to weight changes, meaning scores should not undergo abrupt shifts when weights experience minor perturbations. This smoothness ensures the gradient modulation process remains stable and avoids fluctuations during iterations.

Second, the overall information gain can be approximated as the sum of independent contributions from each parameter. This allows the original global selection problem to be decomposed into a series of parameter-level decisions, enabling the model to operate efficiently even in high dimensions.

##### 4.2.2.1 Optimization modeling of heuristic gating

Building on the decomposability assumption of information gain, we formulate the dynamic activation problem as the following constrained binary optimization:


$$\begin{array}{l} \begin{array}{c} \underset{\text{a}\in {\left\{0,1\right\}}^{\left|\text{W}\right|}}{\max}\;\overset{\left|\text{W}\right|}{\underset{j=1}{\sum }}a_{j}I_{j}\\ \text{ s.t.},\;\overset{\left|\text{W}\right|}{\underset{j=1}{\sum }}a_{j}\leq K, \end{array} \end{array}$$ (12)

where *I*_*j*_ = *I*(**f**; *y*∣*w*_*j*_) denotes the conditional mutual information between feature **f** and class *y* given parameter *w*_*j*_. By introducing a Lagrange multiplier λ≥0, we derive the relaxed objective:


$$\begin{array}{l} J\left(\text{a}\right)=\underset{j}{\sum }a_{j}I_{j}-\lambda \left(\underset{j}{\sum }a_{j}-K\right). \end{array}$$ (13)

Maximizing the objective function after relaxation directly yields a concise activation criterion: a parameter is activated if and only if its information gain exceeds the threshold λ.

This unified threshold rule constitutes the core mechanism of HGF. Its theoretical validity stems from two key points. First, information gain exhibits a monotonic relationship with classification loss, enabling it to reliably reflect parameter importance and serve as a substitute for gradients in decision-making. Second, within the relaxed optimization framework, the threshold λ functions as a Lagrange multiplier to balance parameter importance against activation budget constraints, ensuring each local decision satisfies fundamental optimality conditions.

##### 4.2.2.2 Dynamic threshold adjustment mechanism

To ensure the system adapts to real-time resource availability and satisfies the sparsity constraint |*S*| ≤ *K*, the global threshold τ is dynamically adjusted based on the elapsed processing time. We implement this with an exponential decay strategy:


$$\begin{array}{l} \tau \left(t\right)=\tau_{0}\cdot \exp\left(-\beta \cdot \frac{T_{\text{used}}\left(t\right)}{T_{\max}}\right),\;\left(\beta >0\right). \end{array}$$ (14)

This approach embodies an intuitive trade-off: the system exhibits higher selectivity (with an elevated τ) when computational time is ample and transitions progressively toward inclusiveness (with a reduced τ) as the processing deadline approaches.

A pivotal attribute of this mechanism is its integrated safety net, which prevents excessive sparsity in the subnetwork. In scenarios abundant with low-importance parameters, the threshold τ(*t*) might persist at undesirably high levels, leading to insufficient activations. Our design mitigates this issue through a formal convergence guarantee: by selecting a sufficiently large decay factor β (specifically, β≥ln(τ_0_/τ_min_)), the threshold is assured to decay to a predefined minimum value τ_min_ prior to the deadline *T*_max_. This ensures a baseline number of parameters are perpetually activated, upholding a minimal level of model performance even under adverse conditions.

##### 4.2.2.3 Algorithm implementation

The Heuristic Gating Function (HGF) is elaborated in Algorithm 4. The HGF is engineered to be computationally efficient, intelligent, and resource-aware. Its efficiency predominantly stems from the parallelized importance scoring step (Line 2): leveraging rapid mutual information estimation, scores for all parameters are computed concurrently, achieving *O*(1) complexity in a parallel computing model. To imbue the gating mechanism with resource awareness, we introduce a dynamic threshold (Line 5) that enables real-time adjustment of activation criteria based on the processing deadline.

Algorithm 4 Heuristic gating function (HGF) for dynamic activation. 
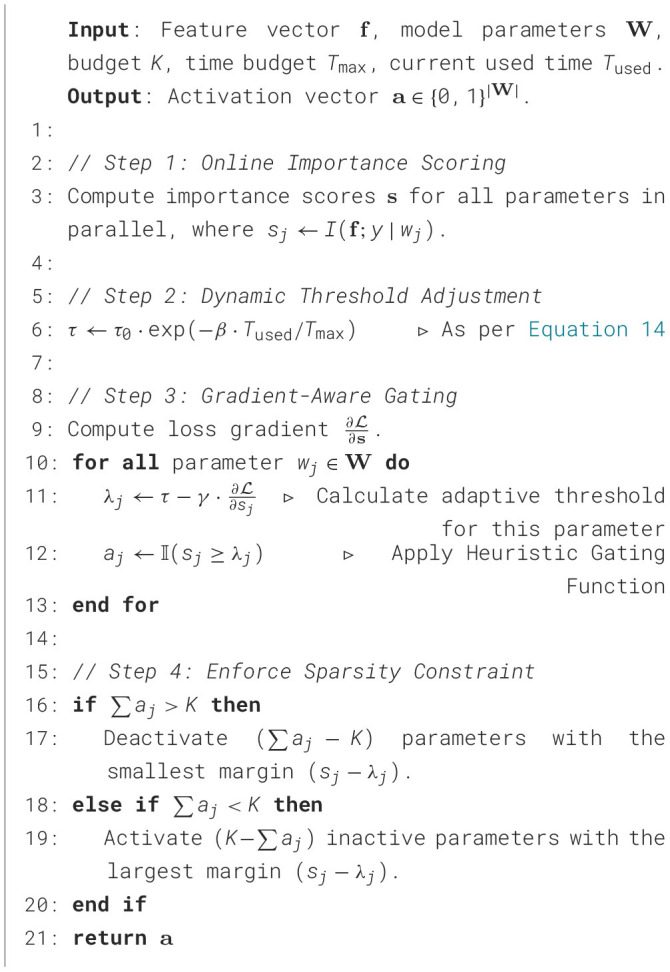

The core intelligence of the HGF resides in its gating decision process (Lines 8–12). Herein, we incorporate a gradient modulation term ($\gamma \cdot \frac{\partial L}{\partial s_{j}}$) to bolster robustness. This design guarantees that if a parameter, despite possessing a high intrinsic score *s*_*j*_, contributes to elevated classification loss, its activation threshold λ_*j*_ is effectively heightened, thereby diminishing its activation likelihood. This furnishes a defense against noisy or deceptive features. Ultimately, to rigorously adhere to the computational budget, we implement a sparsity enforcement step (Lines 15–19): by sorting parameters according to their marginal activation gain (*s*_*j*_−λ_*j*_), this step deterministically executes the most confident activations or deactivations to precisely satisfy the sparsity constraint ∑*a*_*i*_ ≤ *K*.

#### 4.2.3 Dynamic weight adjustment method based on LCNN

The heuristic gating function (Section 4.2.2) enables rapid, feed-forward parameter activation; however, it primarily processes each parameter's activation decision in isolation. To more effectively address the complex, high-dimensional couplings within the feature space, this section introduces a dynamics-based alternative: the Dynamic Weight Adjustment via Local Competitive Neural Network (DWALCNN). The core concept shifts from an independent decision-making framework to a collective one, where parameters in a local neighborhood compete for activation. This competition-collaboration mechanism allows for a more refined parameter selection process, achieving dynamic optimization of weight states with *O*(*K*) complexity.

To formalize this mechanism, the following definitions and assumptions are introduced.

The operational procedure is as follows: For each parameter *w*_*j*_, its local competition domain N(j) is defined as the neighborhood within a radius *r* in the parameter space. Within this domain, the activation state *a*_*j*_ is regulated by a competitive activation function. Specifically, a parameter is activated only if its intrinsic importance score *s*_*j*_ is sufficiently large to overcome the collective inhibitory effect $\underset{k\in N\left(j\right)}{\sum }\mu_{jk}a_{k}^{\left(t\right)}$ from its already-activated neighbors.

To ensure the stability and analyzability of the dynamic system, two fundamental conditions must be satisfied. First, the importance score must smoothly vary with the weight to ensure continuous activation dynamics and convergence. Second, competition must be symmetric and non-self-inhibiting, meaning interactions between parameters should be mutually consistent, and parameters must not inhibit themselves, thereby ensuring a balanced and stable competitive process.

##### 4.2.3.1 LCNN dynamics modeling

We model the evolution of the parameter activation states as a dynamical system. The continuous-time differential equation describing this system is:


$$\begin{array}{l} \frac{da_{j}}{dt}=\sigma \left(s_{j}-\underset{k\in N\left(j\right)}{\sum }\mu_{jk}a_{k}-\tau \right)-\lambda a_{j}, \end{array}$$ (15)

where σ(·) denotes a sigmoidal activation function and λ represents a decay coefficient. This system models the evolution of the activation state for each parameter, driven by its self-importance, inhibition from active neighbors, and natural decay. In practice, the discretized version is utilized.

Before applying LCNN, it is necessary to verify the stability of its dynamical system. Under the previously assumed conditions (smooth importance landscape and symmetric equilibrium of competition coefficients), the update process of LCNN guarantees convergence to a stable equilibrium point.

The core basis for stability stems from constructing a Lyapunov energy function:


$$\begin{array}{l} V\left(\text{a}\right)=-\underset{j}{\sum }\int_{0}^{a_{j}}\sigma^{-1}\left(x\right)dx+\frac{1}{2}\underset{j,k}{\sum }\mu_{jk}a_{j}a_{k}+\tau \underset{j}{\sum }a_{j}. \end{array}$$ (16)

Along the system's trajectory, the time derivative of this function is always non-positive, indicating that the system's energy is continuously decreasing. According to LaSalle's invariance principle (22), the system will ultimately converge to an invariant set satisfying $\frac{dV}{dt}=0$, which corresponds precisely to the equilibrium points of the dynamic equations. Therefore, the activation state of LCNNs must converge stably.

In summary, LCNN maintains a structured activation mechanism while offering a rigorous guarantee of dynamical stability.

##### 4.2.3.2 Competition coefficient learning mechanism

To allow the network to learn which parameters should compete, we design an update rule for the competition coefficients μ_*jk*_ based on Hebbian learning principles (“neurons that fire together, wire together”):


$$\begin{array}{l} \mu_{jk}^{\left(t+1\right)}=\left(1-\eta_{\text{learn}}\right)\mu_{jk}^{\left(t\right)}+\eta_{\text{learn}}\cdot \left(a_{j}^{\left(t\right)}a_{k}^{\left(t\right)}\right), \end{array}$$ (17)

where η_learn_ denotes the learning rate, differing from η which denotes the threshold. This rule enhances competition among frequently co-activated parameters (i.e., increasing μ_*jk*_), thereby promoting the formation of specialized and non-overlapping functional modules.

The Hebbian-based update rule we design (see Equation 17) possesses a crucial intrinsic property—boundedness of coefficients—which is essential for system stability. Since the update rule is essentially a convex combination of values within the interval [0, 1] (i.e., a weighted average of the old coefficient and a new observation), it can be proven by simple induction that if the initial coefficients $\mu_{jk}^{\left(0\right)}$ and the learning rate η_learn_ lie within [0, 1], all subsequent coefficients $\mu_{jk}^{\left(t\right)}$ remain bounded. This property is fundamental to ensure that the learning dynamics of the LCNN do not diverge and remain stable.

##### 4.2.3.3 Algorithm implementation

The complete workflow of the DWALCNN method is presented in Algorithm 5. In the design of DWALCNN (Algorithm 5), we develop a refined dynamics-based activation mechanism as an alternative to the single-shot HGF approach. The core of the algorithm involves simulating the dynamics of LCNN (Lines 9–15) to model competitive interactions among parameters. This competitive process is designed such that a parameter with a high intrinsic score *s*_*j*_ may have its activation suppressed if its active neighbors exert strong inhibition (represented by μ_*jk*_). This enables the network to make more nuanced context-dependent activation decisions by incorporating feature couplings.

Algorithm 5 Dynamic weight adjustment via LCNN (DWALCNN). 
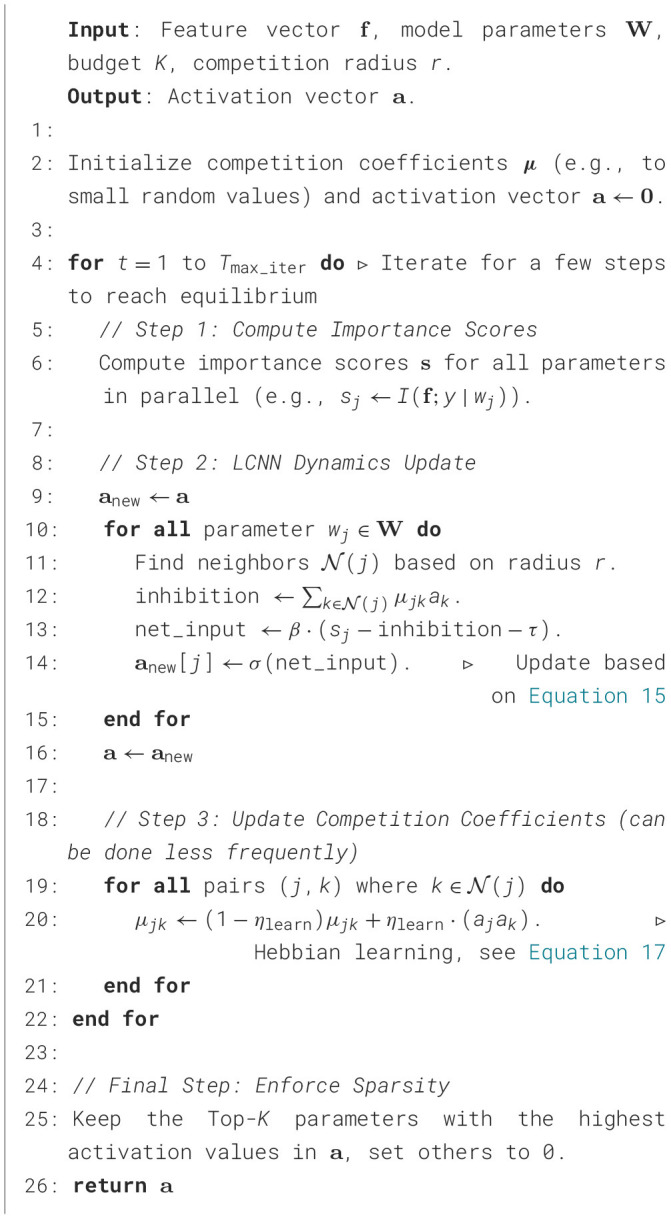

Furthermore, by incorporating a Hebbian learning step (Lines 18–20), we endow the competitive structure with meta-plasticity, allowing it to evolve autonomously. This mechanism enables the competition coefficients to adapt over long timescales, ultimately leading to the emergence of specialized and non-interfering functional modules within the network. The entire process is iterative: the system evolves over multiple steps (*T*_max_iter_) to converge to a steady state, and this hyperparameter allows a trade-off between solution quality and computational latency. Finally, after the dynamical system reaches equilibrium, a sparsity enforcement step is applied (Line 24). This final thresholding converts the continuous activation values into a binary vector, strictly adhering to the sparsity budget *K*, thereby producing an efficient and effective subnetwork for inference.

### 4.3 Online low-complexity incremental learning mechanism

The dynamic subnetwork activation strategies proposed in Section 4.2 enable a degree of adaptability at the sample level. However, real-world data streams often exhibit significant nonstationarity, and relying solely on such local adaptation is insufficient to guarantee long-term stable performance. Over time, data distributions may drift due to factors such as **gradual sensor aging, physiological tremor variations (e.g., user fatigue), or slow shifts in individual behavioral patterns**—a phenomenon known as “concept drift.” Even with dynamic architectures, models with static parameters will gradually become mismatched and degrade in performance.

To this end, this section introduces an online, low-complexity incremental learning framework tailored for resource-constrained edge devices to address the challenge of persistent distributed changes. Crucially, this framework is designed to bridge the gap between short-term anomaly detection and long-term model adaptation. Its core concept involves establishing a rigorous “detect-update” paradigm that supports continuous model evolution while maintaining minimal computational overhead.

The framework operates through two synergistic steps. First, it deploys an efficient recursive CUSUM-based algorithm to precisely identify concept drift occurrences (Section 4.3.1). It is worth noting that while this detection module focuses on identifying deviations from short-term stationarity (acting as a trigger), the continuous application of the subsequent updates enables the model to effectively track long-term, structural concept drift. Upon detection, the framework employs a rapid online learning algorithm, Adaptive Covariance Gradient Descent (ACGD), to facilitate swift model parameter updates (Section 4.3.2). By treating gradual drift as a sequence of incremental statistical shifts, this mechanism allows the decision boundary to continuously evolve. To theoretically ensure long-term reliability, a convergence analysis demonstrates that the combined “CUSUM-ACGD” process is stable and converges almost surely to the optimal parameters for the evolving distribution (Section 4.3.3).

**Figure 4:**
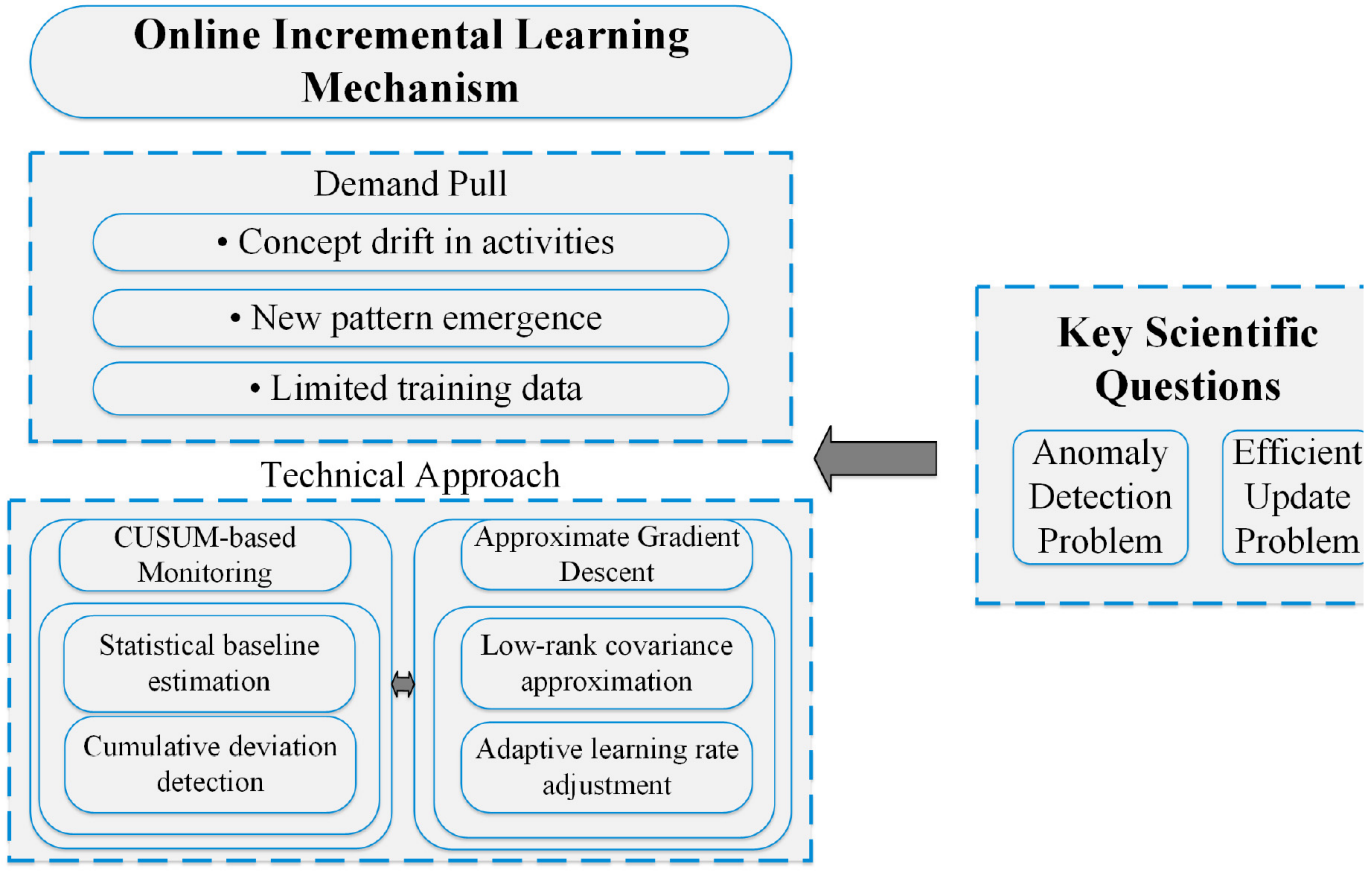
Conceptual framework for the online incremental learning mechanism, outlining the demand pull, key scientific questions, and the proposed technical approach.

The overall framework is conceptualized in Figure 4, highlighting key challenges in nonstationary data environments and decomposing the problem into two core tasks: precise drift detection and efficient online updating. Subsequent sections will detail the design and theoretical foundations of each component.

#### 4.3.1 Short-term stationarity detection method for abnormal behavior features

The core challenge in online learning lies in determining when model updates are truly necessary. Updates should only be triggered when statistically significant changes occur in the data distribution (concept drift). Since abnormalities in venous puncture actions typically occur within < 200 ms, and edge devices require millisecond-level responsiveness, drift detection must be exceptionally efficient. Traditional statistical methods (such as sliding *t*-tests) are unsuitable for this task due to their need for historical data storage (with *O*(*Td*) space complexity) and high computational cost *O*(*dT*^2^) complexity, making them infeasible for real-time applications. This section employs an enhanced CUSUM detection mechanism to perform drift detection with constant-time and constant-space complexity.

The method is based on the assumption that within a short time window, the sequence of features characterizing normal behavior can be regarded as locally stationary, with its mean and covariance remaining approximately constant. When new data deviates from this steady state, an anomaly may occur.

For each feature dimension, we maintain a recursively updated CUSUM statistic:


$$S_{t}^{\left(i\right)}=\max\left(0,\overset{\left(i\right)}{\underset{t-1}{S}}+\frac{f_{t}^{\left(i\right)}-\mu_{0}^{\left(i\right)}}{\sigma_{0}^{\left(i\right)}}-\kappa \right)$$

It accumulates standardized deviations and suppresses minor fluctuations through the parameter κ. When $S_{t}^{\left(i\right)}$ exceeds a preset threshold, drift is detected, thereby ensuring a controlled false alarm rate.

This detection method relies on two key assumptions:

##### 4.3.1.1 Locally stable increment properties

Under normal conditions, the standardized feature increments can be approximated as a martingale sequence, meaning that given all historical information, the expectation of the next increment is zero. This property ensures that the false alarm probability of CUSUM can be rigorously analyzed.

##### 4.3.1.2 Minimum detectable change

When concept drift occurs, the feature mean undergoes at least one quantifiable minimum change (a proportion of the relative standard deviation), defining the smallest drift magnitude detectable by the detector.

Based on this theoretical foundation, the enhanced CUSUM method enables efficient, reliable drift detection suitable for real-time edge devices.

##### 4.3.1.3 Recursive CUSUM detection

To accommodate the strict memory constraints of edge devices, we implement the CUSUM test using recurrence relations. The statistic $S_{t}^{\left(i\right)}$ is updated at each time step, and the baseline parameters ${\hat{\mu }}_{t}$ and ${\hat{\sigma }}_{t}$ are estimated online using an Exponentially Weighted Moving Average (EWMA):


$$\begin{array}{l} {\hat{\mu }}_{t}=\left(1-\alpha_{\text{ewma}}\right){\hat{\mu }}_{t-1}+\alpha_{\text{ewma}}{\text{f}}_{t}, \end{array}$$ (18)

$$\begin{array}{l} {\hat{\sigma }}_{t}^{2}=\left(1-\alpha_{\text{ewma}}\right){\hat{\sigma }}_{t-1}^{2}+\alpha_{\text{ewma}}{\left({\text{f}}_{t}-{\hat{\mu }}_{t}\right)}^{2}, \end{array}$$ (19)

where α_ewma_ is the EWMA smoothing factor. This avoids storing a window of historical data.

Although the recursive implementation achieves exceptional efficiency, its reliability requires theoretical justification. Under two fundamental assumptions: (i) feature increments can be regarded as a martingale sequence during the stable phase; (ii) the magnitude of distribution drift exceeds the detectable threshold, the CUSUM detector guarantees the capture of any significant changes.

When the relaxation parameter is set to κ = δ_min_/2, the CUSUM statistic will cross any finite threshold *h*>0 within a finite time after drift occurs:


$$\mathbb{P}\left(\exists t\leq T^{*}\text{such that }S_{t}^{\left(i\right)}\geq h|\text{shift occurred at }t_{0}\right)=1.$$

Proof based on the Gamma theory (3): Before drift, the expected increment of the statistic is negative, thus keeping it close to zero; after drift, the expected increment becomes positive, causing the statistic to grow at a linear rate until it crosses the threshold. This guarantees a finite expected detection delay and nearly certain detection completion.

##### 4.3.1.4 Adaptive threshold design

To maintain a constant false alarm rate while being sensitive to true changes, the detection threshold *h* must adapt to the estimated variance of the process. We design the threshold as:


$$\begin{array}{l} h_{t}=\Phi^{-1}\left(1-\alpha_{\text{err}}/2d\right)\sqrt{V_{t}} \end{array}$$ (20)

where Φ^−1^ is the inverse of the standard normal CDF, α_err_ is the desired overall false alarm rate, and *d* is the number of features [a Bonferroni correction is applied (23)]. The fundamental principle of the Bonferroni correction method is to distribute the overall significance level α_err_ evenly across each feature dimension, thereby reducing the significance level for each individual dimension to α_err_/2*d*. This ensures that the overall false alarm rate remains controllable in high-dimensional monitoring scenarios.

*V*_*t*_ is the estimated variance of the CUSUM statistic, which for an EWMA process can be approximated as:


$$\begin{array}{l} V_{t}\approx \frac{\alpha_{\text{ewma}}}{2-\alpha_{\text{ewma}}}{\left({\hat{\sigma }}_{t}\right)}^{2} \end{array}$$ (21)

The stability of the adaptive threshold, *h*_*t*_ (see Equation 20), is contingent upon the stability of the process variance estimate. The Exponentially Weighted Moving Average (EWMA) estimator used in this work provides the necessary theoretical guarantees for this condition. A well-established result in statistical process control (24) states that for any smoothing factor α_ewma_∈(0, 1), the variance of an EWMA estimator is bounded. This inherent boundedness ensures that the adaptive threshold *h*_*t*_ is itself stable and bounded, thereby preventing runaway behavior caused by noise or data fluctuations and guaranteeing the long-term reliability of the CUSUM detection mechanism.

##### 4.3.1.5 Algorithm implementation

The complete real-time CUSUM detection process is detailed in Algorithm 6. The algorithm's design was driven by the dual requirements of computational efficiency and statistical robustness for edge deployment. Its efficiency stems from a recursive and memoryless structure. Unlike sliding window approaches, this algorithm stores only the current estimates of the mean, variance, and CUSUM statistic (Line 1), resulting in a minimal space complexity of *O*(*d*). The EWMA (Lines 4–5) and CUSUM statistic updates (Lines 8–9) are performed recursively using computationally inexpensive, element-wise vector operations per time step. This design is critical for real-time processing on resource-constrained devices.

Algorithm 6 Recursive CUSUM detection. 
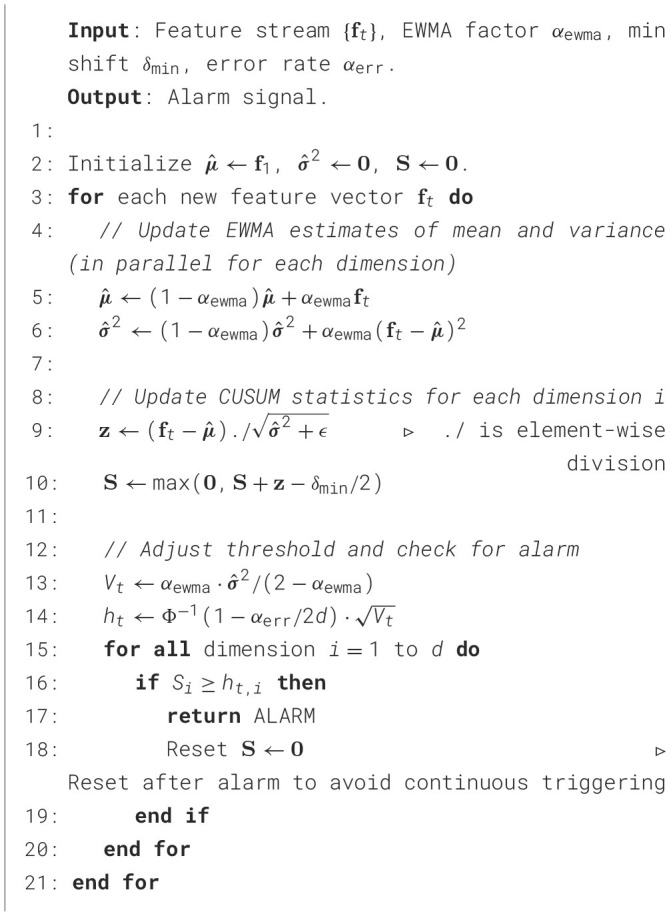

To ensure statistical robustness, the algorithm incorporates an adaptive thresholding and alarm mechanism (Lines 12–18). Rather than using a fixed threshold, which is sensitive to changes in data volatility, the threshold *h*_*t*_ is dynamically recalculated at each step based on the current process variance. This design confers robustness against heteroscedasticity. A Bonferroni correction is applied to the threshold calculation to control the family-wise error rate when monitoring multiple feature dimensions. Upon triggering an alarm, the CUSUM statistic is reset immediately, preparing the system to detect subsequent concept drifts without delay and completing the real-time detection loop.

#### 4.3.2 Fast online model parameter update mechanism construction

Upon receiving an alarm from the CUSUM detector (Section 4.3.1), the system must rapidly update its model parameters to adapt to the detected concept drift. This update process is constrained by two critical requirements: it must be completed within a strict time budget (*T*_update_ ≤ 2 ms) and executed on a resource-limited edge device (typically ≤ 100 MFLOPS). Conventional online learning algorithms are often unsuitable under these constraints; second-order methods, such as Newton's method, which involve computing and inverting the Hessian matrix—an *O*(*d*^3^) operation—are computationally infeasible. Even standard first-order gradient descent can exhibit slow convergence if the loss landscape is ill-conditioned.

To overcome these limitations, this section introduces a novel Adaptive Covariance Gradient Descent (ACGD) algorithm. The foundational concept is to substantially reduce the dimensionality of gradient computations by employing random projection techniques, supported by the Johnson-Lindenstrauss lemma. This methodology constructs a low-rank approximation of the gradient, thereby lowering the computational complexity to *O*(*d*log*d*) while maintaining established theoretical convergence guarantees.

The development and theoretical analysis of the ACGD algorithm rely on a set of formal definitions and assumptions. We begin by formulating the local optimization problem through definitions of the loss function and the dimensionality reduction mechanism. Subsequently, we introduce necessary assumptions regarding the geometric properties of the loss landscape—specifically, strong convexity and smoothness—which form the basis for our convergence analysis.

To formalize the update procedure, we define the local loss function $L_{t}\left(\text{W}\right)$, which constitutes the objective for incremental learning. This function is formulated as the mean squared error over a recent window of anomalous data, augmented with a Tikhonov regularization term to mitigate overfitting. To optimize this loss efficiently, ACGD utilizes a random projection matrix **P**∈ℝ^*r*×*d*^ (where *r*≪*d*), with elements sampled from a standard normal distribution. This matrix enables the projection of the high-dimensional gradient into a lower-dimensional subspace, thus facilitating computationally tractable updates.

The convergence of ACGD depends on two geometric properties of the loss function:

##### 4.3.2.1 Local strong convexity

Within the short adaptation window, the loss function exhibits sufficient curvature in parameter space to ensure the Hessian is bounded near zero, thereby guaranteeing linear convergence.

##### 4.3.2.2 Gradient smoothness

When model parameters change, the gradient variation maintains a smoothness property proportional to the parameter change, enabling a stable and controllable optimization process.

##### 4.3.2.3 Adaptive gradient approximation

To enable fast online updates on resource-constrained edge devices, ACGD avoids computing the full high-dimensional gradient $\nabla L_{t}\left(\text{W}\right)$. Instead, it constructs a low-dimensional approximation using a random projection matrix **P**:


$$\begin{array}{l} {\tilde{\text{g}}}_{t}=\frac{1}{r}{\text{P}}^{T}\left(\nabla L_{t}\left(\text{W}\right)\text{P}\right). \end{array}$$ (22)

This procedure compresses the gradient into an r-dimensional subspace, significantly reducing computational cost while retaining essential geometric information.

The accuracy of this approximation follows from the Johnson–Lindenstrauss (JL) lemma (25), which guarantees that random projection approximately preserves vector norms and inner products. Consequently, the sketched gradient remains close to the true gradient in both magnitude and direction.

Prior theoretical results (26) ensure that when the projection dimension *r* is chosen proportional to ln(*d*), the approximation error remains uniformly bounded with high probability. In particular, the vectorized sketched gradient satisfies:


$$\|\text{vec}\left({\tilde{\text{g}}}_{t}\right)-\text{g}{\|}_{2}\leq \epsilon \|\text{g}{\|}_{2}.$$

This implies that the low-dimensional gradient can serve as a reliable substitute for the true gradient, thereby enabling ACGD to maintain stable convergence behavior even under low-complexity conditions.

##### 4.3.2.4 ACGD algorithm convergence analysis

The parameter update rule for ACGD is a standard gradient descent step, but using the approximated gradient:


$$\begin{array}{l} {\text{W}}_{k+1}={\text{W}}_{k}-\eta_{\text{learn}}{\tilde{\text{g}}}_{k}, \end{array}$$ (23)

where η_learn_ is the learning rate.

Having established the update rule, the central question is whether this iterative process converges to the optimum, especially given the use of an approximated gradient. Under the aforementioned assumptions (including the local strong convexity of the loss function, the Lipschitz continuity of the gradient, and the gradient approximation guarantees based on JL projections), the ACGD iteration process can be proven to converge linearly. Strong convexity ensures that iterations consistently contract toward the optimal point, while smoothness controls perturbations introduced by the approximated gradient.

When the learning rate is set within a conventional range (e.g., η_learn_≈1/*L*), the distance between the current parameters and the optimal solution decreases with each iteration, satisfying the following.


$$\|{\text{W}}_{k+1}-{\text{W}}^{*}{\|}_{F}^{2}\leq \left(1-\frac{\mu }{L{\left(1+\epsilon \right)}^{2}}\right)\|{\text{W}}_{k}-{\text{W}}^{*}{\|}_{F}^{2}.$$

This indicates that the error decays exponentially, with its contraction rate being only slightly affected by the approximate error (ϵ).

Overall, despite using “projected gradients” rather than true gradients, ACGD iterations maintain stability and linear convergence rates consistent with classical gradient descent, reliably converging toward the optimal solution.

##### 4.3.2.5 Algorithm implementation

The implementation of the ACGD algorithm is detailed in Algorithm 7. The design of ACGD is singularly focused on achieving rapid convergence under a minimal computational budget, a critical requirement for online updates at the edge. The cornerstone of its efficiency is the gradient approximation step (Line 7). Unlike traditional second-order methods that require the costly full *d*×*d* Hessian matrix, ACGD performs all essential matrix multiplications within a compressed *r*-dimensional subspace, where *r*≪*d*. This compression is enabled by a one-time random projection (Line 2), which generates a projection matrix **P** that is reused throughout all iterations.

Algorithm 7 Adaptive covariance gradient descent (ACGD). 
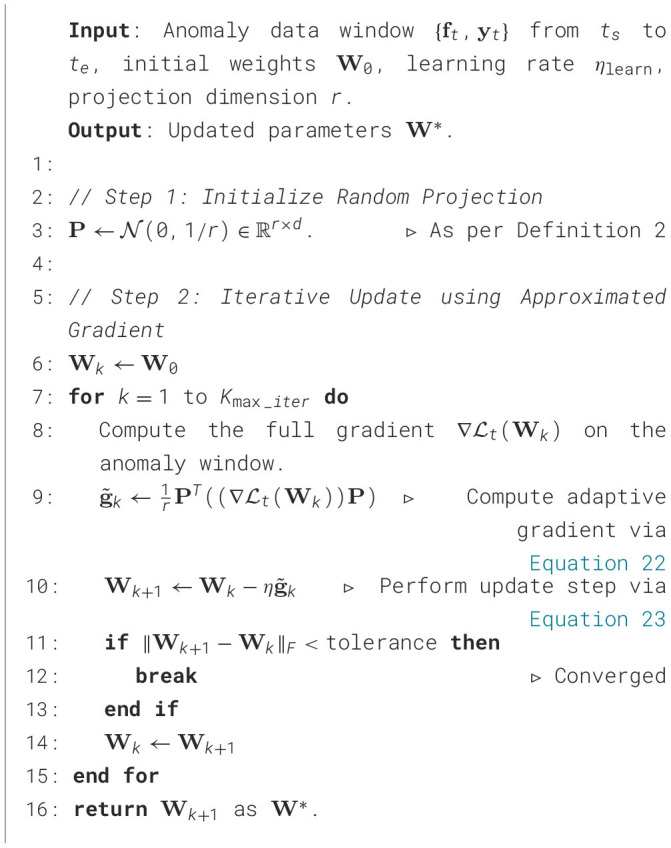

Consequently, the update step (Line 8) remains a simple and computationally inexpensive first-order operation. The key innovation, therefore, lies not in altering the update rule itself, but in drastically reducing the cost of computing a more effective descent direction ${\tilde{\text{g}}}_{k}$. By combining these design choices, ACGD captures essential second-order information—specifically, approximating the action of the Hessian-vector product—without explicitly constructing the Hessian matrix. This approach results in an overall computational complexity of *O*(*d*log*d*), making the algorithm particularly suitable for real-time model updates in resource-constrained edge environments.

#### 4.3.3 Convergence analysis of the online incremental learning mechanism

Having designed the individual mechanisms for drift detection (CUSUM) and rapid parameter adaptation (ACGD), a crucial question remains regarding their integrated behavior: does the combined “detect-then-update” system maintain stability and converge to a meaningful solution over time? This section addresses this question by presenting a convergence analysis for the unified CUSUM-ACGD framework, thereby establishing its long-term reliability.

##### 4.3.3.1 Joint process modeling

The system's evolution is modeled as a stochastic process. The state at time *t* is defined by the tuple *Z*_*t*_ = (*S*_*t*_, **W**_*t*_), where *S*_*t*_ is the CUSUM statistic and **W**_*t*_ is the current weight matrix. This process evolves as a Markov chain, with the transition probability ℙ(*Z*_*t*+1_|*Z*_*t*_) governed by two distinct operational modes. In the default Monitoring Mode, activated when the CUSUM statistic is below its threshold (*S*_*t*_<*h*_*t*_), the model weights remain fixed (**W**_*t*+1_ = **W**_*t*_) while only the statistic *S*_*t*_ is updated. Upon detecting a drift (*S*_*t*_≥*h*_*t*_), the system switches to an update mode. In this mode, the weights are updated via the ACGD algorithm (i.e., **W**_*t*+1_ becomes the output of Algorithm 7), and the CUSUM statistic is reset to zero. The stability of this joint process hinges on demonstrating that updates are triggered sufficiently often and that each update progresses toward the optimal parameter set.

Under drift detection and parameter update conditions, the combined CUSUM–ACGD process guarantees convergence. Specifically, the sequence of parameter estimates {**W**_*t*_} converges almost surely to the optimal parameter matrix W^*^ that tracks the evolving data distribution:


$$\mathbb{P}\left(\underset{t\to \infty }{\lim}\|{\text{W}}_{t}-{\text{W}}_{t}^{*}{\|}_{F}=0\right)=1,$$

where ${\text{W}}_{t}^{*}$ represents the slowly varying optimal parameters for the current concept.

The proof is structured as a three-part argument that synthesizes the results established in the preceding sections.

First, the stability of CUSUM detection has been demonstrated in the preceding text; sustained drift will trigger an alarm within a finite time frame, thereby ensuring timely activation of updates.

Second, the update rules of ACGD ensure that parameter errors contract at a linear rate toward the new local optimum during each update phase.

Finally, by constructing the overall error process as super-martingale and utilizing the Robbins–Siegmund theorem from stochastic approximation theory (27), it can be demonstrated that the overall parametric error converges almost surely to zero.

Collectively, this three-step argument confirms that the CUSUM-ACGD framework constitutes a principled and provably convergent online learning system, aligning with recent theoretical advances in non-stationary online learning (28).

##### 4.3.3.2 Algorithm implementation

The complete, integrated online learning mechanism is summarized in Algorithm 8, which encapsulates the “detect-then-update” loop forming the adaptive core of our method. This algorithm provides a unified framework that integrates the detection and update modules into a single, cohesive process. Central to our design is an event-triggered philosophy, which is essential for ensuring overall system efficiency. At each time step, the system operates primarily in a low-power monitoring state, performing an inexpensive *O*(*d*) detection check for concept drift (Line 7). The computationally intensive ACGD update procedure (Lines 10–13) does not execute on a fixed schedule; rather, it is triggered only when a statistically significant change is detected by the CUSUM module.

Algorithm 8 CUSUM-ACGD online learning framework. 
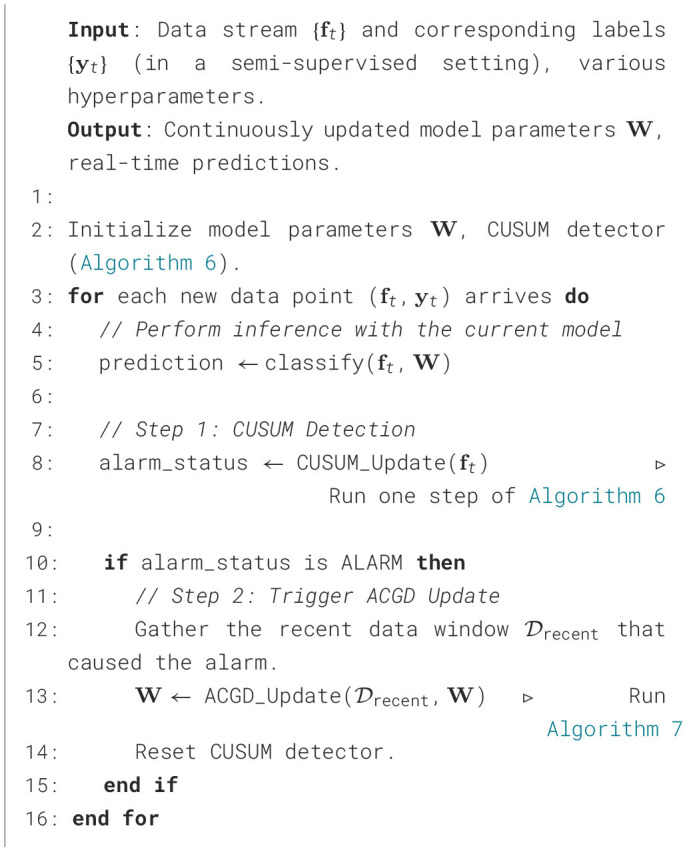

This event-triggered architecture is fundamental for enabling real-time operation on resource-constrained devices. By combining lightweight continuous monitoring with a sporadic yet rapid update mechanism, our framework addresses a core challenge in edge AI: it satisfies strict per-sample latency requirements for real-time inference while maintaining the long-term model adaptation necessary for robust performance in non-stationary environments.

##### 4.3.3.3 Algorithm implementation

Algorithm 8 summarizes the complete, integrated online learning mechanism, encapsulating the “detect-then-update” loop that constitutes the adaptive core of our method. This algorithm provides a unified framework that seamlessly integrates the detection and update modules. The design is founded on an event-triggered philosophy, which is pivotal for achieving overall system efficiency. At each time step, the system primarily remains in a low-power monitoring state, performing an inexpensive *O*(*d*) detection check for concept drift (Line 7). The computationally intensive ACGD update process (Lines 10–13) is not executed periodically but is conditionally triggered only when a statistically significant change is detected by the CUSUM module.

### 4.4 Overall algorithm implementation and optimization strategy at the edge

The preceding sections have introduced the three core computational pillars of our framework: robust feature extraction (Section 4.1), dynamic subnetwork activation (Section 4.2), and online incremental learning (Section 4.3). While each module has been individually optimized, the final system performance depends crucially on their seamless integration into a unified pipeline that can operate reliably on resource-constrained edge devices.

This section addresses this system-level integration challenge. Our goal is to bridge algorithmic design and practical deployment by establishing a coherent execution workflow and a resource-aware control mechanism. To achieve this, we cast the computational load regulation problem as a feedback control problem, enabling the system to maintain stability and responsiveness under dynamic workloads.

The section is structured as follows: first, we outline the overall data and control flow of the integrated method (Section 4.4.1); second, we present the key pseudocode for the main control loop (Section 4.4.2); and third, we detail the design of an adaptive load balancing strategy for dynamically managing computational resources (Section 4.4.3).

#### 4.4.1 Overall flow design of the dynamic subnetwork edge computing method

The real-time resource optimization problem at the edge constitutes a multi-objective dynamic decision problem within a high-dimensional constrained space. The primary challenges are threefold: the dynamic coupling between computational load and task features, the non-linear trade-offs between accuracy and latency/power consumption, and the system uncertainty introduced by incremental learning. Static scheduling or open-loop control strategies are inadequate for addressing these coupled challenges.

Our approach involves constructing a closed-loop coordination mechanism for the dynamic sparse subnetwork, drawing inspiration from state-space modeling in control theory. In this model, the feature vector **f** is treated as the system's state variable, the computational resource configuration parameters (e.g., CPU frequency) serve as the control input, and the classification accuracy and inference latency are the observed outputs. This transformation of the edge computing optimization task into a control system stability and regulation problem represents a key innovation of our design, enabling rigorous analysis and a principled implementation. The overall data and control flow operate as a continuous loop, described in the following paragraphs.

##### 4.4.1.1 Data flow and module interconnection

The process initiates with the raw sensor data stream *X*(*t*), which is first input into the Feature Extraction Module. Operating under the short-term stationarity assumption, this module employs the Fast-PCA algorithm (Algorithm 1) to project the high-dimensional data into a low-dimensional, orthogonal feature vector **f**. The dimensionality d^*^ is adaptively selected by Algorithm 2 to balance information fidelity against computational cost.

Subsequently, this feature vector **f** is passed to the Dynamic Subnetwork Activation Module. This module addresses the NP-hard combinatorial optimization problem using one of the proposed heuristic strategies, selecting an optimal sparse subnetwork defined by weights ${\text{W}}_{c}^{*}$ and performing the classification to produce an output label *y*_*t*_.

Concurrently, the feature stream {**f**_*t*_} is monitored by the Online Incremental Learning Module. The CUSUM detector (Algorithm 6) tests for concept drifts; upon detection, it triggers the ACGD update mechanism (Algorithm 7), which refines the model parameters **W** using recent data. The updated parameters are then propagated to the activation module for subsequent classifications.

##### 4.4.1.2 Closed-loop control flow

The entire process is governed by a resource control loop. Following each inference step, the measured inference time $T_{\text{infer}}\left({\text{W}}_{c}^{*}\right)$ is compared against the target latency *T*_max_. The resulting error signal is processed by a PID (Proportional-Integral-Derivative) controller, whose output adjusts the system's computational resources (e.g., by modulating the CPU/GPU clock frequency) to influence the processing time of the subsequent cycle. This feedback mechanism ensures the system dynamically adapts its resource consumption to remain within the predefined latency budget, thereby constituting a complete, closed-loop adaptive system. This stands in contrast to open-loop systems, which lack the capability to react to unforeseen variations in workload or processing time.

#### 4.4.2 Key algorithm pseudocode design and implementation details

To translate the overall flow design from Section 4.4.1 into a concrete implementation, this section presents the pseudocode for the main control loop of our edge computing framework. Algorithm 9 serves as the master algorithm, orchestrating the interplay between feature extraction, dynamic decision-making, online adaptation, and resource control in each processing cycle.

Algorithm 9 Overall edge framework control loop. 
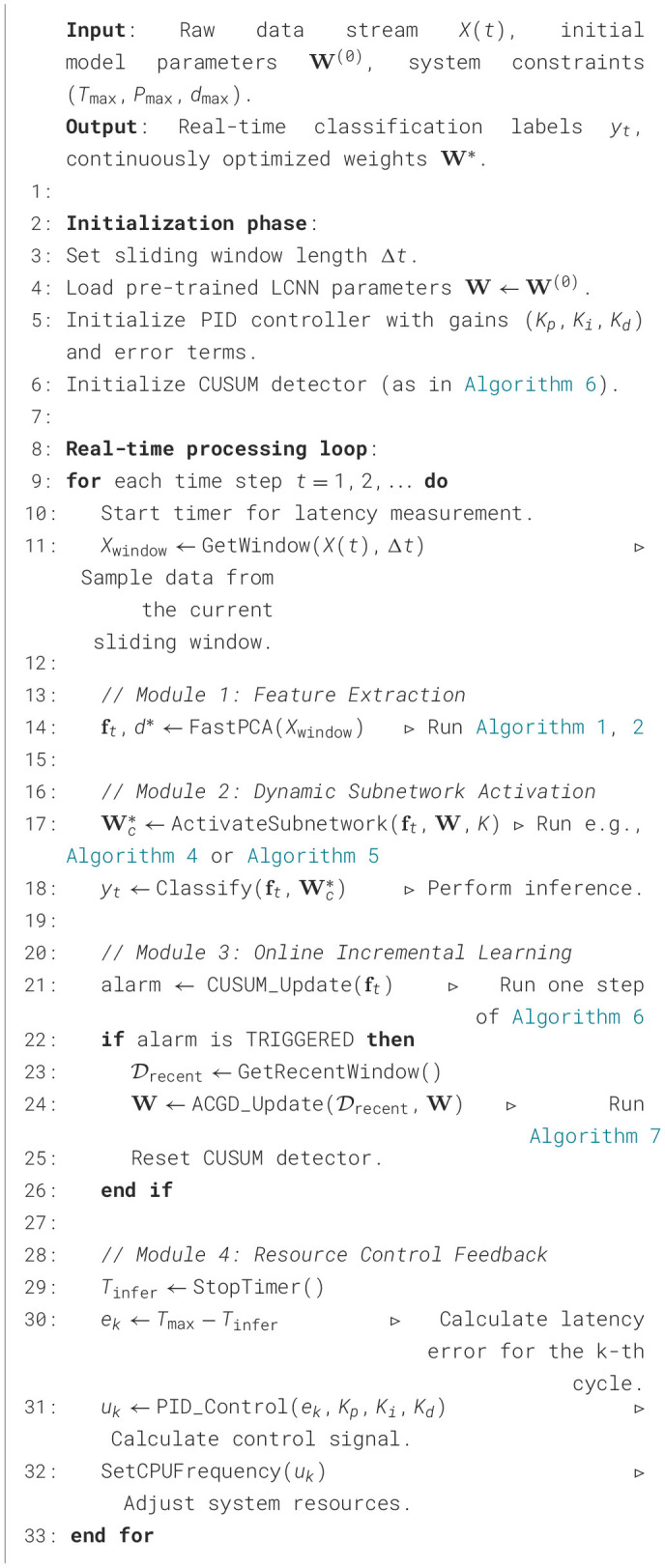

The successful implementation of our master algorithm (Algorithm 9) relies on several key technical choices for its core functions. For the critical task of candidate subnetwork generation (Line 16), we employ a Monte Carlo Tree Search (MCTS) strategy to navigate the vast combinatorial space. This method was selected because it offers a principled approach to balancing exploration—searching for novel and effective structures—and exploitation—leveraging known high-performing structures. The search process is guided by an information gain metric, enabling the “ActivateSubnetwork” function to efficiently identify promising candidate architectures within a constrained time budget.

For CUSUM-based anomaly detection (Line 19), our implementation builds upon the Sequential Probability Ratio Test (SPRT) (3). The update procedure involves computing the log-likelihood ratio:


$$\begin{array}{l} S_{t}=\max\left(0,S_{t-1}+\log\frac{p_{1}\left(y_{t}\right)}{p_{0}\left(y_{t}\right)}\right), \end{array}$$ (24)

where *p*_1_ and *p*_0_ denote the probability density functions under the drift and null hypotheses, respectively. An alarm is raised when the cumulative sum *S*_*t*_ surpasses a predefined threshold *h*, which is carefully calibrated to balance the trade-off between false alarms and missed detections.

The PID control law (Line 27) is implemented in discrete-time form to compute the required resource adjustment *u*_*k*_. The update rule is given by:


$$\begin{array}{l} u_{k}=u_{k-1}+K_{p}\left(e_{k}-e_{k-1}\right)+K_{i}e_{k}\Delta t+K_{d}\frac{e_{k}-2e_{k-1}+e_{k-2}}{\Delta t}, \end{array}$$ (25)

where *e*_*k*_ represents the latency error at cycle *k*. To ensure numerical stability, this formulation is derived from its continuous-time counterpart via the Tustin transformation. The controller gains (*K*_*p*_, *K*_*i*_, *K*_*d*_) are subsequently tuned according to the system's dynamic response characteristics, guided by the stability conditions that will be formally established in the subsequent section.

These implementation decisions are firmly grounded in the theoretical foundations presented earlier. The Fast-PCA projection (Line 13) complies with the optimality conditions set forth. The subnetwork selection process (Line 16) utilizes heuristic methods to approximate solutions to the NP-hard problem, supported by theoretical guarantees on the approximation ratio. Furthermore, the tuning of PID parameters is informed by the stability analysis that will be detailed in Section 4.4.3.

#### 4.4.3 Design of an adaptive load balancing strategy for edge computing

The final component of our framework is a system-level control mechanism designed to dynamically manage computational resources and maintain stable real-time performance. The objective is to continuously adjust resources—such as CPU frequency—to keep the inference latency *T*_infer_ close to a target value *T*_max_, even under fluctuating workloads. This problem can be naturally formulated as a feedback control task.

Although advanced optimal control methods such as Model Predictive Control (MPC) (29) are applicable, their computational demands often render them impractical for real-time deployment on edge devices. To address this, we propose a pragmatic and effective alternative: modeling the system's latency dynamics using an approximate second-order linear model. This simplification allows the use of a classical Proportional-Integral-Derivative (PID) controller, thereby converting the challenging online optimization problem into a more tractable system stabilization task, for which PID control is a well-established and robust solution.

**Figure 5:**
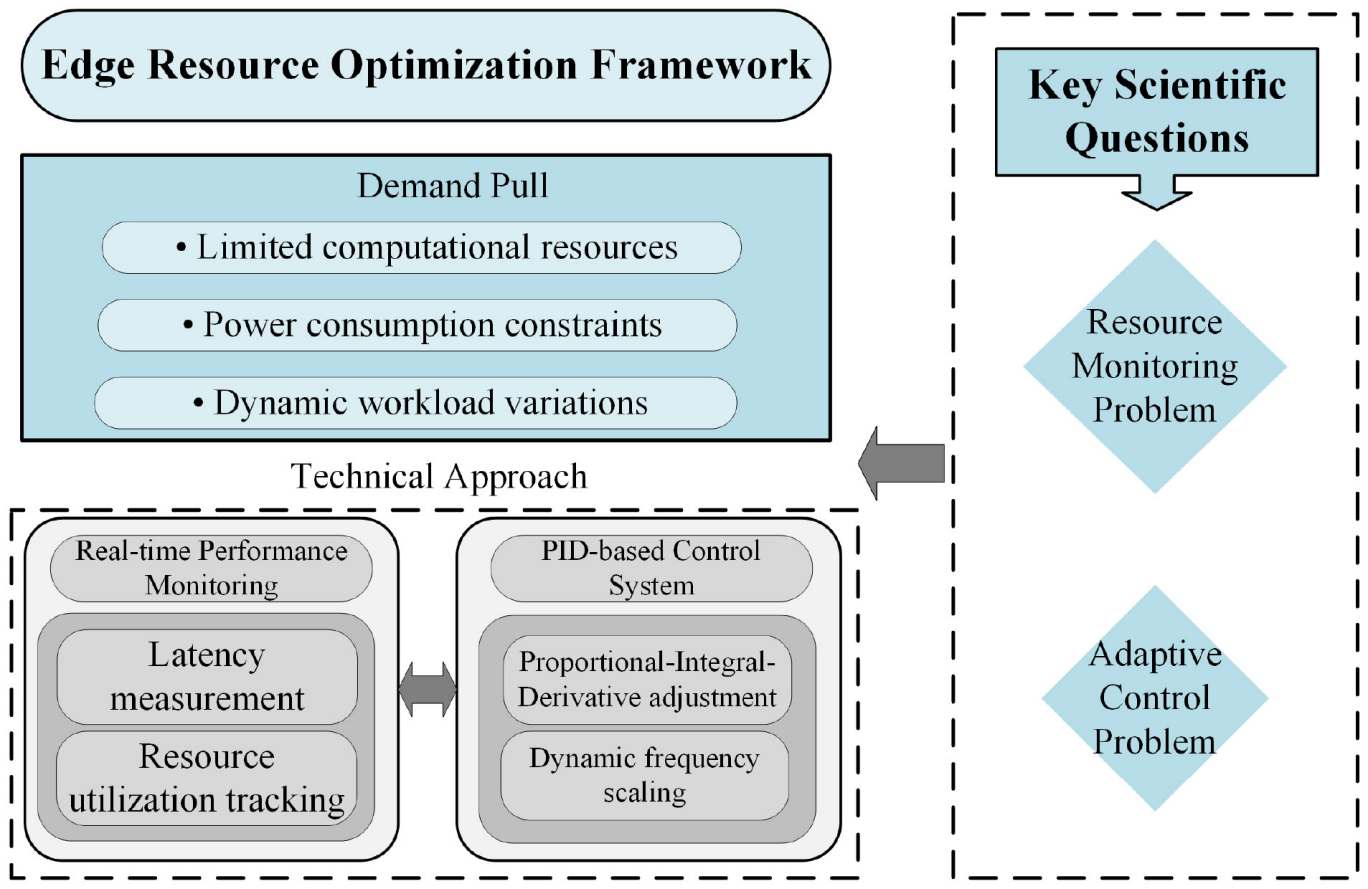
Conceptual framework for the edge resource optimization strategy, outlining the demand pull, key scientific questions, and the proposed technical approach.

The resulting control-theoretic framework is illustrated conceptually in Figure 5. Our solution is structured as a closed-loop system comprising a real-time performance monitoring module, which supplies the feedback signal, and a PID-based control system, which computes the necessary resource adjustments. The following sections elaborate on this model and present a formal stability analysis of the proposed controller.

##### 4.4.3.1 System modeling

We model the dynamics of the inference latency *T*_infer_ as a function of the control input *u*_*k*_ = Δ*f*_CPU_, the change in CPU frequency. The latency at cycle *k*+1 can be approximated by a first-order Taylor expansion around the latency at cycle *k*, plus some process noise *w*_*k*_:


$$T_{\text{infer}}^{\left(k+1\right)}\approx T_{\text{infer}}^{\left(k\right)}+\frac{\partial T_{\text{infer}}}{\partial f_{\text{CPU}}}\Delta f_{\text{CPU}}+w_{k}.$$

Assuming that latency is inversely proportional to frequency (*T*_infer_≈*C*/*f*_CPU_), the partial derivative is $\frac{\partial T_{\text{infer}}}{\partial f_{\text{CPU}}}\approx -C/f_{\text{CPU}}^{2}$. By defining the state vector as the latency error and its derivative, ${\text{x}}_{k}={\left[\Delta T_{k},\Delta {Ṫ}_{k}\right]}^{T}$, we can derive the discrete-time state-space representation of the system:


$$x_{k+1}=\left[\begin{array}{cc} 1 & \delta t\\ 0 & 1-\frac{2C\delta t}{f_{\text{CPU}}^{3}} \end{array}\right]x_{k}+\left[\begin{array}{c} 0\\ -\frac{C\delta t}{f_{\text{CPU}}^{2}} \end{array}\right]u_{k},$$ (26)

where δ*t* is the control period. This state-space model provides the basis for our stability analysis.

With the system formally modeled, the crucial next step is to determine the conditions under which the PID controller can guarantee stability. To this end, we substitute the PID control law into the system model to obtain the corresponding closed-loop characteristic polynomial.

In the continuous-time s-domain, for simplicity of analysis, this equation takes the form:


$$s^{3}+K_{p}^{\prime }s^{2}+\left(K_{i}^{\prime }+A\right)s+K_{d}^{\prime }=0,$$

where *A* is a positive constant related to the system parameters, and $K_{p}^{\prime }$, $K_{i}^{\prime }$, and $K_{d}^{\prime }$ denote the normalized proportional, integral, and derivative gains, respectively.

To determine whether the system is stable, we construct the Routh array for this third-order polynomial based on the Routh–Hurwitz criterion (30). According to the construction results, the four entries in the first column of the Routh array are:


$$1,K_{p}^{\prime },\frac{K_{p}^{\prime }\left(K_{i}^{\prime }+A\right)-K_{d}^{\prime }}{K_{p}^{\prime }},K_{d}^{\prime }.$$

According to the Routh–Hurwitz criterion, the system is stable if and only if all the above terms are positive. This yields the necessary and sufficient condition for the stability of a PID controller:


$$K_{p}^{\prime }>0,\;K_{d}^{\prime }>0,\;K_{p}^{\prime }\left(K_{i}^{\prime }+A\right)>K_{d}^{\prime }$$

The third inequality defines the feasible range for the integral gain $K_{i}^{\prime }$:


$$K_{i}^{\prime }>\frac{K_{d}^{\prime }}{K_{p}^{\prime }}-A$$

Provided that the PID gain satisfies the aforementioned three conditions, the closed-loop system can avoid oscillations and maintain stable dynamic response.

##### 4.4.3.2 PID controller design for robustness

Although DVFS-based latency regulation forms a hybrid, mildly nonlinear feedback loop due to discrete frequency levels, actuator bounds, and workload/measurement disturbances. In our framework, the PID controller is not used to control the nonlinear learning dynamics; it regulates only the scalar inference latency.

The PID is implemented in discrete time using the incremental form in Equation 25, consistent with DVFS updates executed once per control period Δ*t* (i.e., the sampling interval of the resource control loop). For analysis, we consider an operating region where the latency–frequency relation is monotonic and can be approximated by a locally linearizable form (e.g., *T*_infer_≈*T*_0_+*C*/*f*_CPU_). Under this local approximation, the closed-loop characteristic equation admits sufficient stability conditions via the Routh–Hurwitz criterion, which guides gain selection away from oscillatory regimes.

To improve robustness under noise and to prevent frequent switching among discrete DVFS levels, we employ (i) **bounded control action** enforced by the DVFS range and (ii) **EWMA low-pass filtering** of the measured latency before forming the error signal. In addition, common implementation refinements such as deadband/hysteresis, rate limiting, and integrator anti-windup can be enabled to further suppress chattering under high-frequency measurement noise.

##### 4.4.3.3 Auxiliary optimization strategies

In addition to the core PID control, we employ two auxiliary strategies to further enhance the robustness and efficiency of the resource management.

**Dynamic priority scheduling:** In scenarios with multiple concurrent tasks, not all tasks are equally important. We introduce a dynamic priority weight ω_*i*_ for each task *i*, which could be based on its deadline or importance: ω_*i*_ = exp(−μ·delay_*i*_). The total available resource *f*_total_ is then allocated proportionally:


$$f_{i}=\frac{\omega_{i}}{\sum_{j}\omega_{j}}f_{\text{total}}.$$

This ensures that more critical tasks receive a larger share of the computational resources.

**Resource reservation:** To handle sudden bursts in workload or unexpected processing demands, a portion of the total resources is always kept in reserve. We set a hard threshold *f*_reserve_ = ρ*f*_total_, where the reservation factor ρ can be dynamically adjusted based on historical load statistics. This reserved capacity acts as a buffer, improving the system's resilience to transient spikes in demand.

## 5 Experiments

This section presents a systematic evaluation of the proposed framework across multiple datasets. The study methodically examines its effectiveness, computational efficiency, and robustness. The evaluation includes comparisons with four representative human activity recognition baselines (CNN, DeepConvLSTM, iSPLInception, and HART), ablation studies, and multi-dimensional performance analyses. The results demonstrate a superior trade-off between accuracy and efficiency and stable adaptation under non-stationary conditions.

### 5.1 Experimental design and setup

This subsection describes the experimental configuration, the construction of five heterogeneous datasets, the choice of four representative HAR baselines, a unified set of evaluation metrics, and a standardized runtime environment. The complete setup ensures fair comparison and reproducibility.

#### 5.1.1 Datasets

To assess generalization and practical value, evaluation is performed on multiple sources and scales, including three simulated datasets (30K/50K/100K) and two real-world benchmarks (UCI, MotionSense, HHAR, and Real World), as detailed in Table 1.

**Table 1:** Overview of datasets used in the experiments.

**Dataset**	**Number of classes**	**Feature dimension**	**Sensor type**	**Sampling rate**
100K Dataset				
50K Dataset	6	9	Acc, Gyro, Mag	60 Hz
30K Dataset				
HCI_HAR Dataset	6	6	Acc, Gyro	50 Hz
RealWorld Dataset	8	9	Acc, Gyro, Mag	50 Hz

##### 5.1.1.1 Venipuncture simulation dataset

Three venipuncture datasets are constructed at different scales, including 100K, 50K, and 30K. The base streams are generated with high-fidelity physical modeling. Each sample contains nine-axis inertial measurement unit (IMU) sensor data, including three-axis accelerometer, three-axis gyroscope, and three-axis magnetometer, sampled at 60 Hz. Six operation classes are included: approaching, aiming, searching, stabilizing, puncture, tremor.

The simulation uses multi-layer physical models designed to introduce specific types of concept drift. Physiological tremor is modeled by a combination of harmonic and modulation components, simulating variations in user fatigue over time. Mechanical response is modeled with a bimodal exponential function. Sensor noise includes gradual sensor drift (modeled as random walk), cross-axis interference, and environmental noise. Parameters are randomized within ranges to mimic operator and environment variability.

##### 5.1.1.2 HCI dataset

HCI is a standard benchmark for HAR (31). It contains daily activities from 30 subjects. The dataset uses a six axis sensor configuration with a three axis accelerometer and a three axis gyroscope. Six activities are included, namely walking, stair up, stair down, sitting, standing, and lying. This dataset supports comparison with prior work on a common task.

##### 5.1.1.3 RealWorld dataset

RealWorld HAR is a large dataset collected in natural environments (32). It contains eight activities from 15 subjects. In this study, only the nine axis IMU channels are used, including gyroscope, accelerometer, and magnetometer. Only the sensor worn at the abdomen is selected for analysis. This setting targets lightweight deployment on edge devices and matches the constraint of a single sensor in resource limited scenarios. Multi sensor fusion increases communication and compute cost, which is beyond the scope here. Crucially, the data exhibit significant long-term drift, which primarily stems from changes in sensor placement (displacement during activity), individual behavioral variability (inter-subject and intra-subject variability), and environmental noise fluctuations. This dataset is used to assess robustness and generalization under real deployment conditions.

##### 5.1.1.4 Data preprocessing

All datasets use Z score normalization and sliding windows for sample segmentation. The window size is 128 samples, the stride is 64 samples, and the overlap is 50%. For datasets with fewer channels, zero padding or feature selection is applied to match a nine dimensional input.

#### 5.1.2 Baselines and time complexity analysis

Four representative HAR deep learning models are selected as baselines. The models cover different architectural families and support a clear comparison. Training and testing use a unified hardware environment and consistent settings.

**CNN** is a classic convolutional neural network for HAR (9). It uses two one-dimensional convolution layers for feature extraction. Each layer is followed by batch normalization and ReLU. Global average pooling reduces dimensionality, and a fully connected layer performs classification. The inference time complexity is approximately $O\left(\underset{l}{\sum }\left(K_{l}\cdot C_{in,l}\cdot C_{out,l}\right)+C\cdot d_{global}\right)$.

**DeepConvLSTM** combines convolutional layers with long short-term memory (LSTM) layers (10). Four one-dimensional convolution layers extract hierarchical features. The features are then processed by two LSTM layers for temporal modeling. The inference complexity is approximately $O\left(T\cdot \underset{l}{\sum }\left(K_{l}\cdot C_{in,l}\cdot C_{out,l}\right)+T\cdot H^{2}\right)$, where *H* is the LSTM hidden size.

**iSPLInception** builds on an Inception ResNet style architecture for sensor-based HAR (11). It uses multi scale convolution kernels to capture features at different time scales. Residual connections ease training. Depthwise separable convolutions reduce compute. The inference complexity is approximately $O\left(T\cdot \underset{scale}{\sum }\left(K_{scale}\cdot C_{in}\cdot C_{out}\right)\right)$.

**HART** is a transformer-based model for HAR (12). It uses six Transformer encoder layers with self-attention to capture long-range temporal dependencies. Position encoding and sensor embeddings are combined. The main complexity comes from attention with *O*(*T*^2^·*d*).

The proposed adaptive edge inference framework integrates several components. The total complexity reflects combined costs. Fast PCA has complexity *O*(*d*^2^). Dynamic sparse subnetwork selection has complexity *O*(*K*·*C*·*d*·*T*). CUSUM detection has *O*(*d*). The PID controller has *O*(1). In total, the time complexity is approximately *O*(*K*·*C*·*d*·*T*+*d*^2^). Sparse activation and incremental updates keep practical runtime within acceptable limits.

#### 5.1.3 Evaluation metrics

A unified set of evaluation metrics is used for fair comparison across all methods.

- **Accuracy**. The ratio of correct predictions to all samples.
- **Per sample time (ms)**. The average time to process a single sample in milliseconds. For the adaptive method this includes feature extraction, subnetwork activation, and any online learning trigger.

#### 5.1.4 Experimental environment

Experiments were conducted on an 11th Gen Intel Core i7-11370H CPU at 3.30 GHz with 16 GB RAM. The software environment utilizes Windows 11 and Python 3.8.

Regarding the baseline methods, all models are trained using the Adam optimizer with an initial learning rate of 0.001, a batch size of 32, and for 100 epochs. The dataset split is 70% training, 20% validation, and 10% testing. We primarily adopted the default parameter settings as recommended in their respective original literature. However, to ensure a fair comparison, we also performed a limited grid search for key hyperparameters on the validation set to guarantee optimal convergence on our specific datasets.

##### 5.1.4.1 Model architecture and hyperparameters

Key hyperparameters for the proposed method were determined through empirical validation using a grid search strategy on a separate validation set. Specifically, the selection criterion prioritized configurations that offer an optimal trade-off between recognition accuracy and computational efficiency, strictly adhering to the latency constraints of edge devices.

To improve reproducibility, the proposed model architecture (backbone used by the dynamic sparse activation module) and the key hyperparameters used in the experiments are summarized in Tables 2, 3. All hyperparameters in Table 3 are fixed across all reported experiments unless stated otherwise.

**Table 2:** Proposed model architecture (lightweight 1D CNN backbone).

**Block**	**Channels**	**Kernel/stride**	**Output**
Input window	9	–	9 × 128
Conv1D+BN+ReLU+MaxPool	64	5/1 + MaxPool(*k* = 2, *s* = 2)	64 × 64
Conv1D+BN+ReLU+MaxPool	128	5/1 + MaxPool(*k* = 2, *s* = 2)	128 × 32
Conv1D+BN+ReLU	256	5/1	256 × 32
AdaptiveAvgPool1D + FC	256 → *C*	–	logits in ℝ^*C*^

**Table 3:** Key hyperparameters and experimental settings.

**Item**	**Value**
Windowing	window size 128, stride 64 (50% overlap)
Normalization	Z-score per channel
Train/val/test split	70% / 20% / 10%
Optimizer	Adam
Learning rate	0.001
Batch size	32
Epochs	100
Fast-PCA oversampling	*p* = 5
Adaptive dimension selection	η_min_ = 0.9, δ = 0.05
Sparsity budget	*K* = 5
Online update step size	η_learn_ = 0.001
CUSUM parameters	α_ewma_ = 0.1, (δ_min_, α_err_) = (2.0, 0.005)
PID gains	*K*_*p*_ = 0.5, *K*_*i*_ = 0.1, *K*_*d*_ = 0.05
Runtime measurement	per-sample time includes feature extraction + subnetwork activation + any online-learning trigger

### 5.2 Main results and analysis

This subsection presents a comprehensive comparison across five datasets and four baseline models. The results verify strong performance and generalization under different data sources and scales.

#### 5.2.1 Performance comparison across datasets

**Figure 6:**
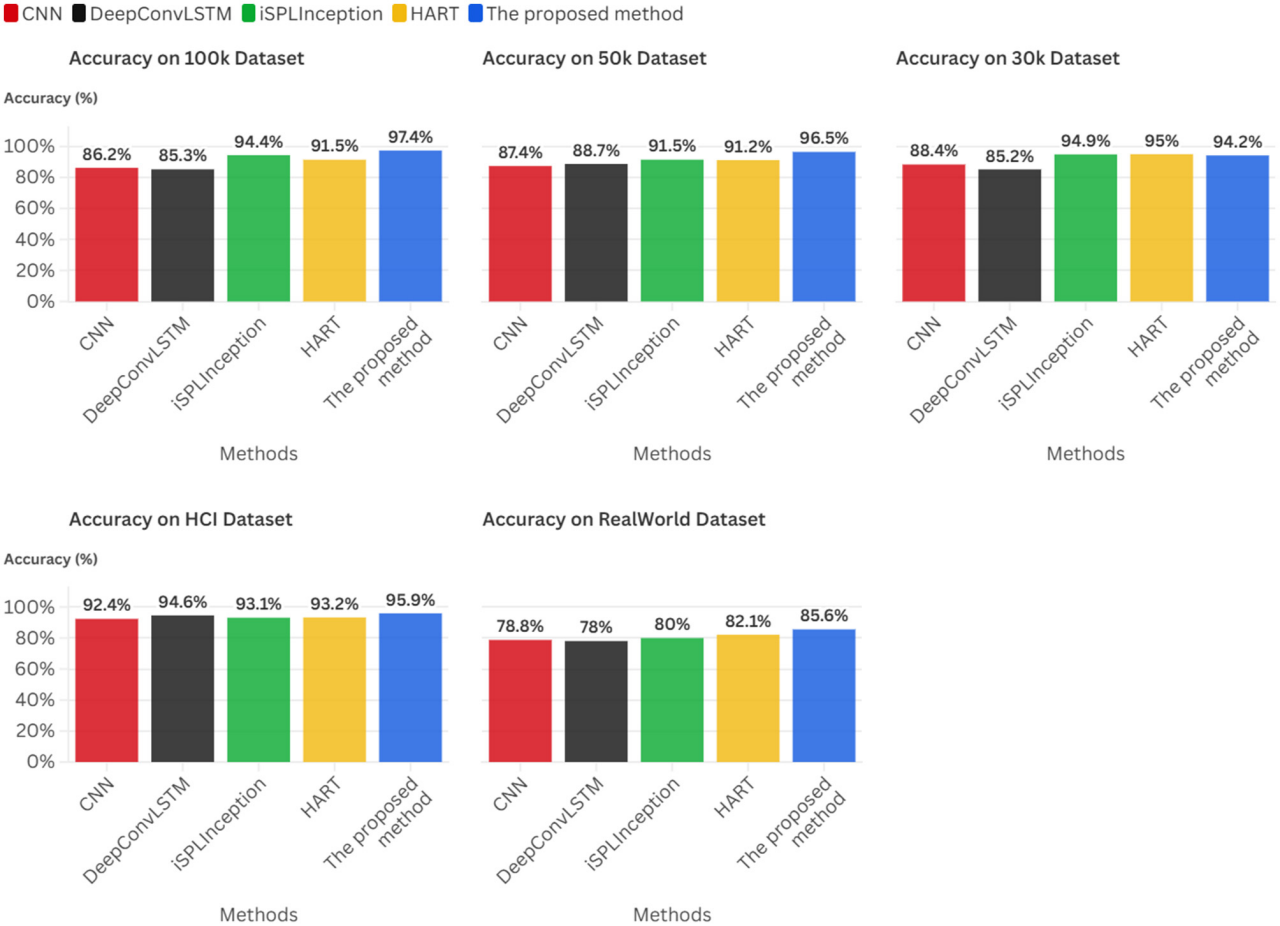
Accuracy comparison across five datasets.

As reported in Table 4 and Figure 6, the proposed method's accuracy was evaluated across five datasets. The proposed method achieves the highest accuracy on four datasets, including 100K, 50K, HCI, and RealWorld. On the 30K dataset, its accuracy of 94.2% is marginally below that of HART (95.0%), marking a minor gap of 0.8 percentage points. Relative to the best baseline, gains on 100K and 50K are 3.0 and 7.8 percentage points. Gains on HCI and RealWorld are 1.3 and 3.5 percentage points. Inference latency remains between 0.89 and 1.14 ms across all datasets, and is lower than complex deep models such as iSPLInception at 3.45 to 3.67 ms and HART at 2.42 to 3.21 ms.

**Table 4:** Accuracy comparison across five datasets (%).

**Model**	**100K dataset**	**50K dataset**	**30K dataset**	**HCI**	**RealWorld**
CNN	86.2	87.4	88.4	92.4	78.8
DeepConvLSTM	85.3	88.7	85.2	94.6	78.0
iSPLInception	94.4	91.5	94.9	93.1	80.0
HART	91.5	91.2	95.0	93.2	82.1
Proposed method	97.4	96.5	94.2	95.9	85.6

On the venipuncture simulation datasets, the method shows clear advantages on 100K and 50K. As dataset size grows from 30K to 100K the advantage over iSPLInception increases from negative 0.7 to positive 3.0 percentage points. This pattern suggests that the incremental learning mechanism benefits from richer data and matches the convergence analysis in Section 4.3. The relative weakness on 30K may come from limited samples for robust mutual information estimation within the subnetwork selection.

On the HCI benchmark the accuracy reaches 95.9%, surpassing DeepConvLSTM at 94.6% and HART at 93.2%. On the RealWorld dataset the accuracy is 85.6%, an improvement of 3.5 percentage points over HART. RealWorld includes environmental noise, sensor drift, and subject variability. These results underscore the model's robustness under nonstationary conditions.

From an efficiency perspective, latency remains between 0.89 and 1.14 ms on all datasets, well below the 16.7 ms bound for 60 Hz processing. Relative to iSPLInception at 3.56 ms the reduction is about 69%. Relative to DeepConvLSTM at 1.51 ms the reduction is about 26%. Although CNN reaches 0.53 ms, the accuracy is 11.2 percentage points lower.

##### 5.2.1.1 Sources of performance advantages

The gains arise from three components working together. Fast PCA adjusts the feature dimension according to the data, reducing noise while preserving key information. Orthogonal feature decoupling reduces the dimension from nine to about three to five while maintaining about 95% of total variance. Dynamic sparse subnetwork selection activates parameters according to input complexity. The average activation rate is about 35% of all parameters. The online incremental learning mechanism with CUSUM detection and ACGD updates adapts to distribution changes and maintains high accuracy. These effects are consistent with the theory in Sections 4.1, 4.2.

#### 5.2.2 Efficiency analysis

Efficiency is a key factor for real time edge deployment. The method achieves a balanced point between accuracy and latency and meets the timing requirement.

##### 5.2.2.1 Latency comparison

**Figure 7:**
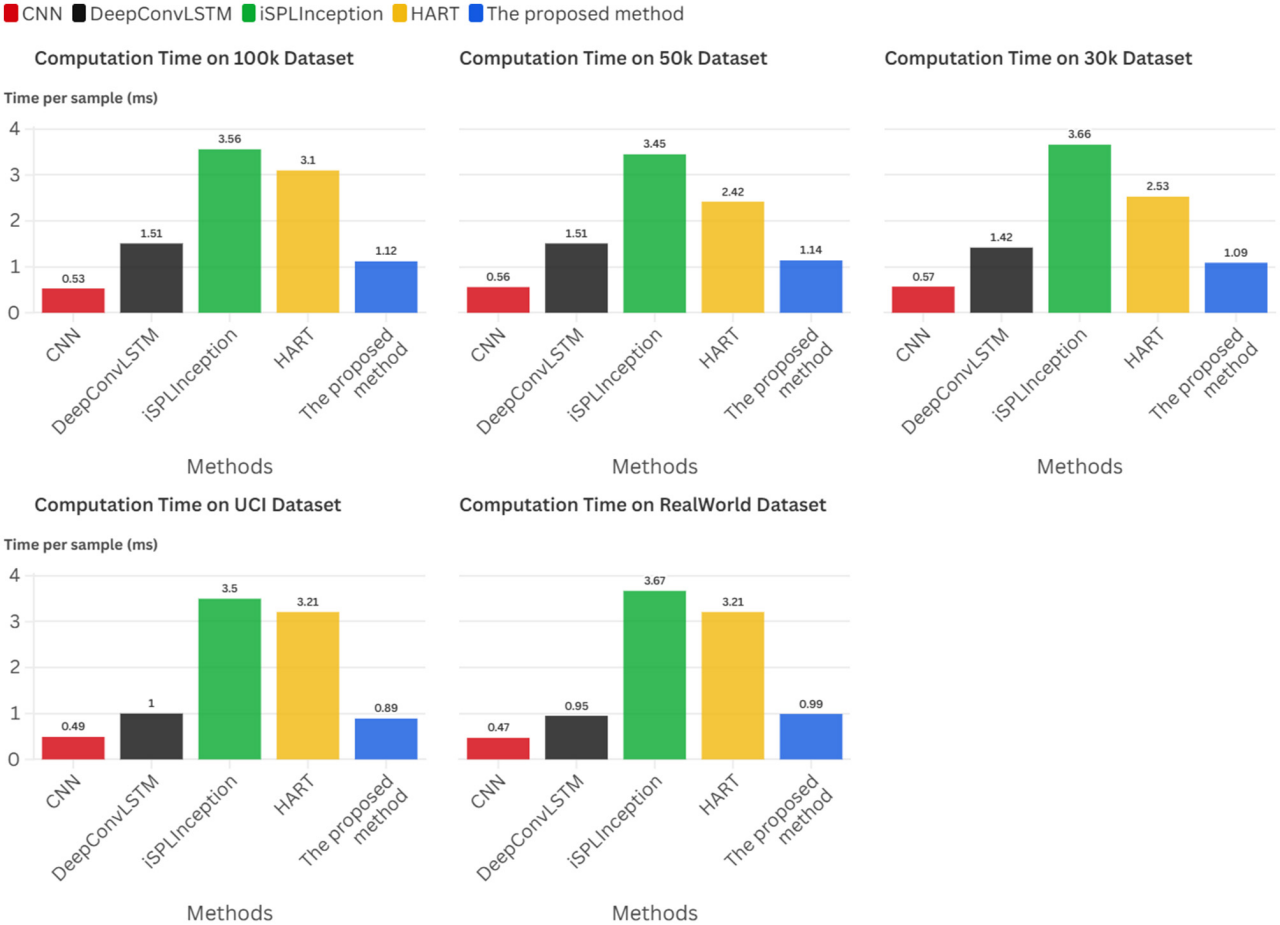
Per sample inference time comparison across five datasets.

Patterns are clear across model families, as shown in Table 5 and Figure 7. Lightweight CNN models are fastest with 0.47 to 0.57 ms, due to a simple two layer design with complexity *O*(*K*·*C*·*d*). This speed comes with a notable accuracy drop. iSPLInception has higher cost from multi scale parallel convolutions with 3.45 to 3.67 ms and complexity $O\left(T\cdot \underset{scale}{\sum }\left(K_{scale}\cdot C\right)\right)$. HART uses attention with complexity *O*(*T*^2^·*d*) and time of 2.42 to 3.21 ms. DeepConvLSTM reaches 0.95 to 1.51 ms, reflecting a balance between local feature extraction and temporal modeling but with *O*(*T*·*H*^2^) cost.

**Table 5:** Per sample inference time across five datasets (ms).

**Model**	**100K dataset**	**50K dataset**	**30K dataset**	**HCI**	**RealWorld**
CNN	0.53	0.56	0.57	0.49	0.47
DeepConvLSTM	1.51	1.51	1.42	1.00	0.95
iSPLInception	3.56	3.45	3.66	3.50	3.67
HART	3.10	2.42	2.53	3.21	3.21
Proposed method	1.12	1.14	1.09	0.89	0.99

The proposed method keeps latency between 0.89 and 1.14 ms. First, the values meet the 60 Hz timing bound, leaving headroom for system scheduling. Second, latency is stable across datasets with a standard deviation of 0.09 ms, showing robustness to changes in data characteristics. Third, relative to complex models the reduction is large. The main source is sparse activation that avoids redundant full network compute.

#### 5.2.3 Accuracy and efficiency trade off analysis

Pareto optimality provides a clear view of the position on the accuracy and latency plane. On the 100K dataset the proposed method reaches 97.4% accuracy and 1.12 ms latency. Relative to iSPLInception at 94.4% and 3.56 ms, accuracy improves by 3.0 percentage points and latency reduces by about 69%. Relative to CNN at 86.2% and 0.53 ms, the additional 0.59 ms increases latency by about 111% but gains 11.2 percentage points in accuracy. A simple utility function *U* = α·Acc−(1−α)·*T* shows that for α>0.05 the proposed method is strictly better than CNN. Relative to DeepConvLSTM at 85.3% and 1.51 ms, accuracy improves by 12.1 percentage points while latency drops by about 26%.

Gains come from three coordinated mechanisms. Fast PCA lowers the feature complexity from *O*(*n*·*d*) to *O*(*n*·d^*^) with d^*^≈0.4*d*. Dynamic sparse selection activates only part of the parameters with constraint ∑*a*_*i*_ ≤ *K*. Online learning maintains high accuracy under concept drift and avoids performance decay present in static models. The approximation ratio in Section 4.2 is not below 1 − 1/*e*, which supports near optimal performance.

#### 5.2.4 System stability and disturbance rejection analysis

**Figure 8:**
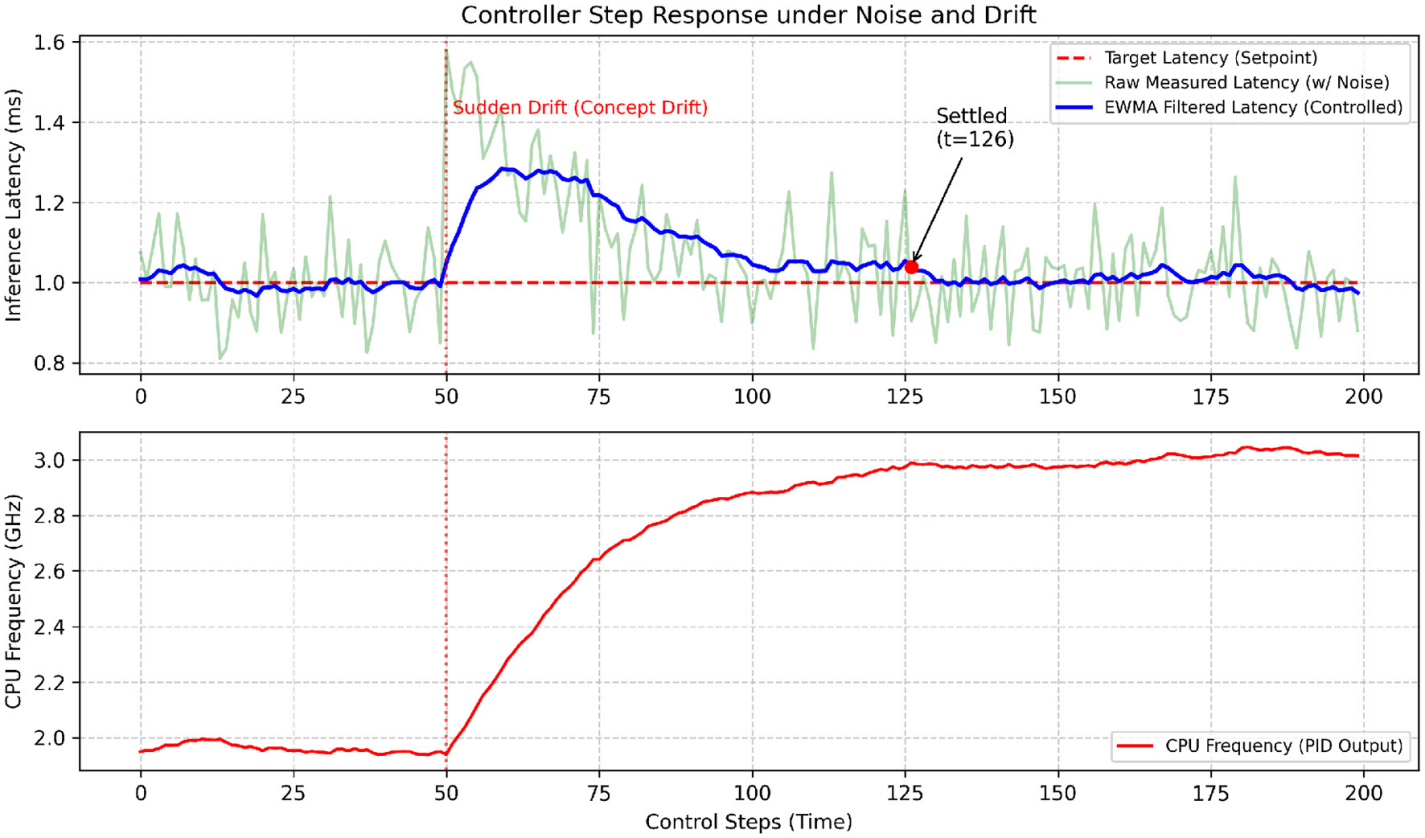
Numerical stress test validating closed-loop control stability under extreme edge cases.

To rigorously validate the safety of the resource control mechanism in clinical settings, we conducted a numerical stress test based on the closed-loop dynamics modeled in Equation 26. The simulation utilized the exact PID gains (*K*_*p*_ = 0.5, *K*_*i*_ = 0.1, *K*_*d*_ = 0.05) and the EWMA filtering parameter (α = 0.1) defined in our experimental setup. Figure 8 illustrates the system's step response under two concurrent stress conditions: high-frequency sensor noise (σ = 0.1) and a sudden +50% workload surge simulating abrupt concept drift.

Quantitative analysis yields the following performance metrics:

- **Settling time:** Adopting the standard 5% criterion, the system restabilizes within **76 control cycles** (approx. 1.2s) following the massive workload drift.
- **Overshoot:** The maximum overshoot is contained at **28.38%**. The EWMA filter effectively dampens the initial physical impact, preventing secondary oscillatory overshoot.
- **Steady-state error:** The integral action eliminates residual error, maintaining a steady-state precision of **0.01680 ms** (< 2% deviation), ensuring strict adherence to the real-time deadline.

These results confirm that the proposed control strategy maintains robust stability without oscillation, even under extreme edge cases.

### 5.3 Ablation study

This subsection is dedicated to quantifying the contribution of each module (summary results are provided in Table 6). Fast PCA, dynamic sparse subnetwork selection, and online learning are removed in turn. Changes in accuracy and latency are analyzed and the combined effect is reported.

**Table 6:** Accuracy comparison in ablation experiments across five datasets (%).

**Configuration**	**100K dataset**	**50K dataset**	**30K dataset**	**HCI**	**RealWorld**
w/o Fast PCA	97.0	95.1	94.5	95.0	83.8
w/o CUSUM & Online learning	87.7	85.5	87.9	91.2	81.2
w/o candidate selection	98.2	97.6	95.2	96.6	85.0
Proposed method	97.3	96.4	94.2	95.9	85.6

#### 5.3.1 Contribution of fast PCA

**Figure 9:**
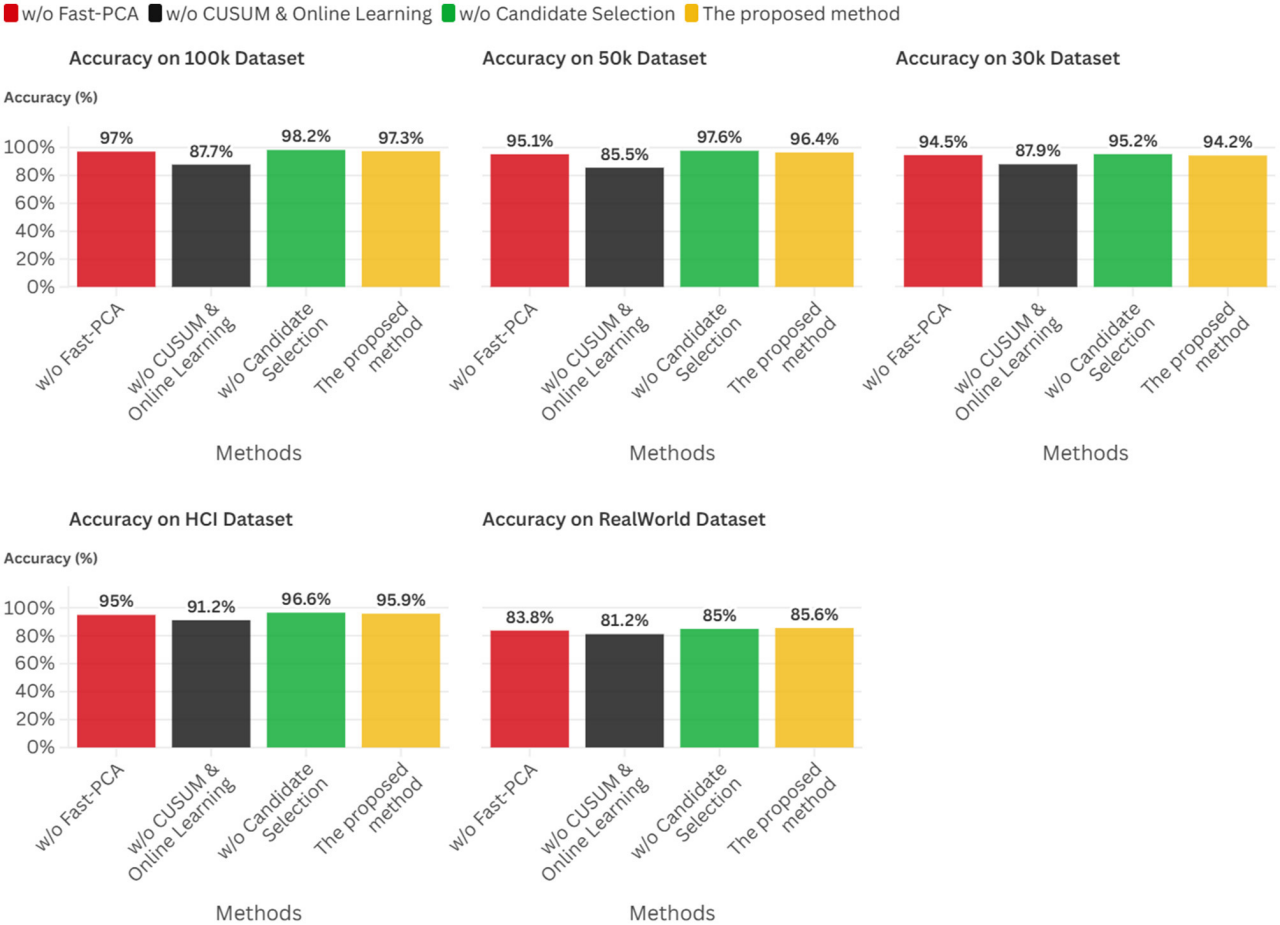
Accuracy comparison in ablation experiments across five datasets.

The removal of Fast PCA changes accuracy differently across datasets, as visualized in Figure 9. On 100K accuracy drops from 97.3% to 97.0%. On RealWorld it drops from 85.6% to 83.8%. On 30K it slightly increases from 94.2% to 94.5%. Two factors explain the overall pattern. First, raw nine dimensional sensor data have redundancy and noise, and feeding them directly can reduce discriminative power. Second, the random projection theory in Section 4.1 shows that Fast PCA preserves about 95% of variance and filters secondary noise components, improving the signal to noise ratio of features.

**Figure 10:**
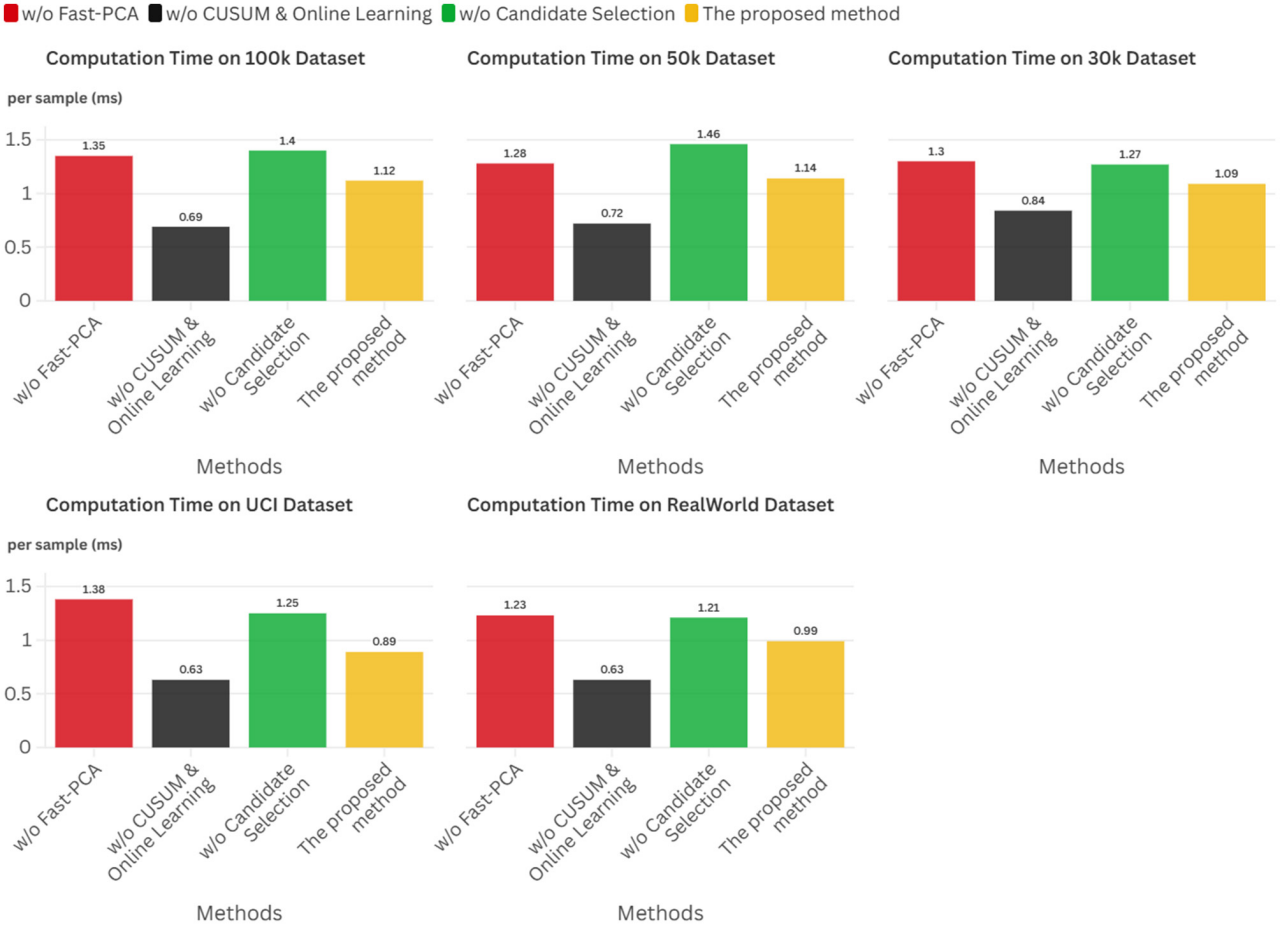
Per sample inference time in ablation experiments across five datasets.

Removing Fast PCA increases computation time by about 21%, as illustrated in Figure 10. On 100K latency rises from 1.12 ms to 1.35 ms. Three effects explain this pattern. Although Fast PCA adds front end compute, it reduces the feature dimension from nine to about three to five. This lowers the complexity of subnetwork selection and classification from *O*(|**W**|·*d*) to *O*(|**W**|·d^*^) with d^*^≈4. Orthogonal decoupling reduces redundancy and lowers the condition number of the covariance matrix. Higher quality features improve mutual information estimates in the gating function, which reduces invalid parameter activation and avoids redundant compute paths. Together these effects reduce overall cost.

#### 5.3.2 Dynamic sparse subnetwork selection

Removing dynamic selection and using the full network gives a small accuracy increase on some datasets with gains up to about 1.2 percentage points. This shows that the full network can capture more features under some distributions. The increase comes with a notable latency cost of about 25%.

The trade off is asymmetric. Relative to dynamic selection, accuracy increases by about 1.0 percentage points while latency increases by about 0.28 ms. In cost and benefit terms, each 1% accuracy gain costs about 0.28 ms latency. The rise from 1.12 ms to 1.40 ms approaches real time limits. In the resource management framework in Section 4.4, under 60 Hz processing a 0.28 ms increase does not break the bound but reduces headroom and limits concurrency. More importantly, dynamic selection adjusts compute according to input complexity and provides flexibility under uncertain workloads on edge devices.

Efficiency gains come from two sources. Parameter level sparsity activates only part of the network according to the constraint ∑*a*_*i*_ ≤ *K*. Sample adaptive control adjusts activation rate according to input complexity. According to Section 4.2, this strategy provides a locally optimal solution to the constrained problem in Equation 12.

Note that our “per-sample time” metric is end-to-end; for the proposed method it includes feature extraction, candidate selection, and any triggered online-learning update. Therefore, the reported latency already accounts for the selection overhead. As shown in Table 7, disabling candidate selection increases latency from 1.12 ms to 1.40 ms on the 100K dataset, and similarly degrades latency on other datasets. This indicates that the selection overhead is more than offset by the computation saved through sparse activation, yielding a net runtime benefit.

**Table 7:** Per sample inference time in ablation experiments across five datasets (ms).

**Configuration**	**100K dataset**	**50K dataset**	**30K dataset**	**HCI**	**RealWorld**
w/o Fast PCA	1.35	1.28	1.30	1.38	1.23
w/o CUSUM & Online learning	0.69	0.72	0.84	0.63	0.63
w/o candidate selection	1.40	1.46	1.27	1.25	1.21
Proposed method	1.12	1.14	1.09	0.89	0.99

#### 5.3.3 Online learning mechanism

Removing CUSUM detection and ACGD updates gives the largest accuracy drop, with an average reduction of 7.2 percentage points across datasets. This confirms the key role of online learning for long term robustness. Two factors explain the need. Both simulation and real datasets include concept drift, where statistics change across operation stages. Static models cannot adapt. According to Section 4.3, the CUSUM and ACGD combination converges to parameters that track the evolving distribution, which supports long term performance.

Removing online learning lowers latency by about 38% because detection and updates are no longer computed. The efficiency gain comes with a strong accuracy cost. On 100K accuracy drops by 9.6 percentage points from 97.3% to 87.7%, which is 6.7 percentage points below iSPLInception at 94.4%. This shows the value of online adaptation under nonstationary data. Static models run faster but their accuracy decays over time. From a deployment view the additional 0.43 ms remains within the real time bound of 16.7 ms and yields a 9.6 percentage point accuracy gain. Under nonstationary streams, the benefit from adaptation is larger than the cost.

### 5.4 Discussion

Results support generalization on three dimensions. Across data sources the method maintains advantage on both simulation and real sensor data, which suggests that adaptation does not depend on one generator. Across data scales performance improves steadily from 30K to 100K samples, which suggests robustness to training size. Across application scenarios differences between nursing operations and daily activities do not change relative performance, which suggests that the framework is domain independent.

On RealWorld the performance is notable. The dataset includes nonstationarity from sensor drift, intra class variability from subjects, and noise from environment. Under these strict conditions the method still outperforms HART, a Transformer based baseline. This shows that a lightweight architecture with adaptive mechanisms is effective under both resource limits and nonstationary data.

### 5.5 Limitations and future work

Several limitations remain. The CUSUM-based change detection is reactive and needs enough observations before triggering updates. Sudden distribution changes may cause a delay. System performance depends on hyperparameters including PID gains, CUSUM parameters, and projection dimension. Values are set by validation in this study, but may need adjustment in other scenarios. Behavior on larger datasets and longer time spans is not yet validated. The cumulative effect of online learning and long-term stability of CUSUM statistics need further study.

Future directions include predictive drift detection to trigger pre-adjustment, automatic online hyperparameter tuning, and support for multimodal sensor fusion. Extending the framework to edge and cloud collaboration can balance latency and compute by splitting tasks between real-time inference on the edge and periodic refinement in the cloud. Hardware-specific acceleration that uses random projection and sparse compute can further lower latency for stricter real-time conditions.

## 6 Conclusion

This paper addresses a problem at the intersection of applied mathematics and edge computing: the real-time classification of high-dimensional, non-stationary stochastic processes under stringent computational constraints. We establish that traditional methods, which rely on empirical risk minimization over static datasets, are theoretically ill-suited for such dynamic tasks due to their inherent violation of the stationarity assumption. To overcome this limitation, we propose a novel framework that integrates principles from stochastic signal processing, combinatorial optimization, and online learning theory. This framework synergistically combines a Fast-PCA variant for dimensionality reduction, a heuristic-based strategy to address the NP-hard problem of dynamic subnetwork activation, and a CUSUM mechanism for low-complexity, convergent parameter updates.

Experimental results corroborate the theoretical advantages of the framework. Across five datasets exhibiting significant concept drift, the proposed method achieves accuracies ranging from 85.6% to 97.4%. On the RealWorld dataset, which represents a real-world application scenario, the method attains an accuracy of 85.6%, outperforming the Transformer-based HART (82.1%) by 3.5 percentage points. This is achieved while maintaining an inference latency of approximately 1.0 ms, comfortably meeting the real-time requirement (16.7 ms threshold). These findings demonstrate that the method surpasses existing baselines in both accuracy and computational efficiency, striking an effective trade-off between adaptability and performance. This validates that the synergistic design of a lightweight architecture and an adaptive mechanism can overcome the limitations of traditional static models in nonstationary environments.

In conclusion, this work contributes a new methodological framework for designing adaptive intelligent systems in resource-constrained settings. Its primary contribution lies in the establishment of a unified, closed-loop system that provides a rigorous theoretical foundation for each core component, from feature extraction to model adaptation. The resulting solution is both robust and scalable, with significant potential for application in other domains challenged by dynamic data streams, such as financial time-series analysis or the Industrial Internet of Things. Future research will focus on the theoretical analysis of more sophisticated, predictive drift detection mechanisms to further advance the state-of-the-art in online adaptive learning.

## Data Availability

The raw data supporting the conclusions of this article will be made available by the authors, without undue reservation.
